# Comprehensive Blood Metabolome and Exposome Analysis, Annotation, and Interpretation in E-Waste Workers

**DOI:** 10.3390/metabo14120671

**Published:** 2024-12-02

**Authors:** Zhiqiang Pang, Charles Viau, Julius N. Fobil, Niladri Basu, Jianguo Xia

**Affiliations:** 1 Faculty of Agricultural and Environmental Sciences, McGill University, Ste-Anne-de-Bellevue, QC H9X 3V9, Canada; zhiqiang.pang@mail.mcgill.ca (Z.P.);; 2 School of Public Health, University of Ghana, Legon, Accra P.O. Box LG 13, Ghana; 3 West Africa Center for Global Environmental & Occupational Health, College of Health Sciences, Legon, Accra P.O. Box LG 13, Ghana

**Keywords:** e-waste, metabolomics, exposomics, metals, mass spectrometry

## Abstract

**Background:** Electronic and electrical waste (e-waste) production has emerged to be of global environmental public health concern. E-waste workers, who are frequently exposed to hazardous chemicals through occupational activities, face considerable health risks. **Methods:** To investigate the metabolic and exposomic changes in these workers, we analyzed whole blood samples from 100 male e-waste workers and 49 controls from the GEOHealth II project (2017–2018 in Accra, Ghana) using LC-MS/MS. A specialized computational workflow was established for exposomics data analysis, incorporating two curated reference libraries for metabolome and exposome profiling. Two feature detection algorithms, *asari* and *centWave*, were applied. **Results:** In comparison to *centWave*, *asari* showed better sensitivity in detecting MS features, particularly at trace levels. Principal component analysis demonstrated distinct metabolic profiles between e-waste workers and controls, revealing significant disruptions in key metabolic pathways, including steroid hormone biosynthesis, drug metabolism, bile acid biosynthesis, vitamin metabolism, and prostaglandin biosynthesis. Correlation analyses linked metal exposures to alterations in hundreds to thousands of metabolic features. Functional enrichment analysis highlighted significant perturbations in pathways related to liver function, vitamin metabolism, linoleate metabolism, and dynorphin signaling, with the latter being observed for the first time in e-waste workers. **Conclusions:** This study provides new insights into the biological impact of prolonged metal exposure in e-waste workers.

## 1. Introduction

Exposomics is an emerging field of study that aims to comprehensively investigate environmental exposures and their associations with health outcomes [1,2,3]. A significant bottleneck in exposome research lies in the measurement and characterization of exposomes [4,5]. Liquid chromatography–tandem mass spectrometry (LC-MS/MS)-based untargeted metabolomics is widely employed in exposomics studies [6,7]. However, downstream spectra processing, statistical analysis, and functional interpretation remain challenging tasks. A comprehensive yet easy-to-use computational workflow is urgently needed to help advance this nascent research field.

Electronic and electrical waste (e-waste) has become a significant global challenge [8,9,10]. Millions of tons of e-waste are exported annually to developing countries, where large industries have been established to extract valuable materials through e-waste collection and recycling [8,11]. Individuals working or living in these e-waste processing regions face high exposure to hazardous chemical components released during the handling of e-waste. Since metal contaminants are typically non-biodegradable [11], persistent organic pollutants and heavy metals are found at significantly high levels in those exposed to e-waste [12,13]. These exposures have been associated with adverse health outcomes such as DNA damage [14], endocrine disruption [15], and impaired neurodevelopment [16]. A comprehensive study of the heavy metal exposure and associated metabolic responses of e-waste workers is needed to further our understanding in this area.

High-resolution LC-MS/MS spectral processing remains a challenging task. Over the past few decades, different algorithms have been developed to address this issue [17,18,19,20]. One widely used algorithm is *centWave*, which employs continuous wavelet transformation and Gaussian fitting to detect and filter MS features [18]. While effective, this method requires the optimization of many parameters to achieve optimal results, leading to the development of an auto-optimized workflow to streamline this process [21]. In addition, trace-level features often do not yield sufficient data points for modeling Gaussian shape, potentially limiting *centWave*’s effectiveness in processing exposomics spectra, where many exposures are expected to occur at a very low level. More recently, an alternative algorithm, *asari*, has been introduced [22]. Unlike *centWave*, *asari* focuses on the cumulative shape of features across multiple samples, rather than individual peak shapes. This approach makes it particularly suitable to detect trace-level features. While both *centWave* and *asari* have demonstrated potential for exposomics feature detection, their applications in this domain still require comprehensive benchmarking to determine their efficacy in detecting features in complex exposomics datasets.

This study aims to investigate the blood exposome and metabolome from e-waste workers. We first developed a computational workflow for exposomics data analysis. We performed an untargeted LC-MS/MS analysis to characterize both the metabolome and exposome in whole blood samples from 100 e-waste workers from Agbogbloshie, a well-known e-waste processing site, and 49 non-e-waste workers as controls from Madina Zongo; both locations are in Accra, Ghana [23]. This manuscript describes the exposomics data analysis workflow and its application in the study of e-waste workers, aiming to uncover associations between heavy metal exposure and biological dysfunctions as well as the broader biological impacts of such exposures.

## 2. Materials and Methods

### 2.1. Participants Recruitment and Ethic Assessment

Participants were drawn from the GEOHealth II cohort situated in Accra, Ghana as described previously [24,25,26,27,28,29,30]. In brief, a community durbar was conducted to inform and familiarize eligible participants with the study’s objectives and procedures. The inclusion criteria for participants from Agbogbloshie (e-waste site) included adult males aged 18 years and above who have worked at the e-waste site for at least 6 months, while subjects from Madina Zongo (control) were age-matched with similar cultural characteristics as e-waste worker with respect to culture and had lived in the Madina Zongo for at least 6 months. Madina Zongo (control) site is about 18 km from the e-waste site. Participants were included between March 2017 and October 2018. This study was approved by the Research Ethics Office of McGill University (Study ID: A05-M26-16B) and the Ethical and Protocol Review Committee at the College of Health Sciences, University of Ghana (protocol identification number CHS-ET/M.4-P 3.9/2015–2016).

### 2.2. Sample Collection and Processing

Blood collection and metals analysis were detailed previously [24]. In brief, 10 mL of whole venous blood was drawn from each participant by a phlebotomist into EDTA trace-elements-free tubes. Blood was stored at the University of Ghana at −80 °C, and later shipped on dry ice to McGill University (Montreal, QC, Canada) for metals analysis.

For metabolomics measures, blood samples were thawed on ice for one hour and subsequently vortexed for 30 s. For preprocessing, 100 µL of each sample was transferred to a 1.5 mL Eppendorf microcentrifuge tube, following the established protocol described previously [31]. After filtration, 150 µL of each processed sample was transferred into LC-MS vials, fitted with 250 µL glass inserts, in preparation for elution with chromatography and mass spectrometry acquisition. A long-term reference sample was prepared from a standard serum sample (Sigma-Aldrich, Sigma, St. Louis, MO, USA).

### 2.3. Quantification of Blood Metals

Metals were analyzed as per the methods detailed earlier [24]. Blood was digested in nitric acid and then analyzed by inductively coupled plasma mass spectrometer (ICPMS Varian; 820 MS, SpectraLab Scientific Inc. Markham, ON, Canada).

### 2.4. LC-MS/MS Assay

All chemicals were purchased with the details listed below. Ammonium acetate (NH4AC) was purchased from Sigma-Aldrich (Sigma, St. Louis, MO, USA). Acetonitrile (ACN), methanol (MeOH), 0.1% formic acid (FA) in water, 0.1% FA in ACN, and pure water were purchased from Fisher Chemical (Morris Plains, NJ, USA).

The LC-MS and MS/MS assays were conducted using a UHPLC system (Thermo Scientific™ UltiMate™ 3000 System, Waltham, MA, USA). For the separations, a hydrophobic C18 column was employed for reverse-phase liquid chromatography, and a hydrophilic interaction liquid chromatography (HILIC) column was used, as previously described [31]. LC-MS1 acquisition was sequentially performed for each mode (C18-ESI^+^, C18-ESI^−^, HILIC-ESI^+^, HILIC-ESI^−^). Upon completing the LC-MS1 acquisition, immediate data analysis was conducted to generate all LC-MS features as targets for data-dependent acquisition (DDA) of MS2 spectra, with a set window size of 0.4 Da. Instrumental settings for untargeted DDA and sequential window acquisition of all theoretical fragment ion mass spectra (SWATH-MS) data-independent acquisition (DIA) were same as the previous study [31].

The long-term reference samples were injected alongside the entire sample injection process in order to monitor the stability of the whole system and correct the potential signal drift or batch effects.

### 2.5. Spectral Data Pre-Processing

Raw MS data were converted and centroided with MSconvert, ProteoWizard (v3.0.23068) [32] into mzML format for data pre-processing. MetaboAnalystR 4.0 was used to optimize the parameters of the *centWave* algorithm [21]. Both *asari* and *centWave* were used to perform MS1 feature detection to generate a peak table. The ppm was set to 5 for *asari* while keeping other parameters at default. All optimized parameters for *centWave* were summarized in Supplementary Table S1.

MS2 data processing including both DDA and DIA data processing were completed with MetaboAnalystR 4.0 based on the feature tables generated by *asari* or *centWave*, respectively. The MS2 similarity was evaluated based on the dot-product method [20]. The matching score threshold was set at 80/100. The complete MS2 library was used to identify as many chemical candidates as possible for both algorithms. This spectral reference library contains more than 10.4 million reference spectra for 1.5 million compounds [31,33].

### 2.6. Database Curation

Metabolome and exposome libraries were curated in prior LC-MS/MS experiments and data processing of the e-waste workers’ dataset. The libraries were established based on current publicly available academic databases. Specifically, the Metabolome library was curated directly from the Human Metabolome Database (HMDB, v5.0) [34]. The exposome library was manually curated from multiple public databases, including the KEGG Drug database [35], Microbial Metabolites Database [36], Toxin-Toxin-Target Database (T3DB) [37], FooDB (www.foodb.ca, accessed on 10 September 2024), Phenol-Explorer [38], Exposome-Explorer [39], and NORMAN Suspect List Exchange database [40]. All compounds were labelled and identified with their InChIKeys for both the metabolome and exposome libraries. The metabolome universe consists of 217,875 compounds, while the exposome universe contains 253,045 compounds. Compounds in the exposome library were further categorized into 21 classes based on information from the NORMAN Suspect List Exchange database [40] and the source of each compound. Compounds from different categories were labeled with their InChIKeys. These InChIKeys were used for the chemical classification of compounds identified based on MS2 spectra.

### 2.7. Bioinformatic and Statistical Analysis

Bioinformatic analyses were conducted using MetaboAnalystR 4.0 [31] and MetaboAnalyst 6.0 [33], incorporating *mummichog*-based functional analysis, principal component analysis (PCA), t-tests, and fold change analysis. Dose–response was performed with a workflow from the *protti* R package, as described previously [41]. The entire datasets were normalized with log-transformation before dose–response analysis. All additional statistical analyses, including Pearson’s correlation and linear regression analyses [42], were performed using the basic statistical packages available in R (v. 4.4.1). Statistical significance was accepted at *p* < 0.05, unless otherwise specified.

## 3. Results

### 3.1. Characteristics of Subjects

A total of 100 e-waste workers and 49 age-matched controls were studied from March 2017 to October 2018 as part of the GEOHealth II study described by Takyi et al. [26,27,30]. The demographic characteristics of all participants are summarized in Table 1. Statistical analysis of various characteristics, including body mass index (BMI), smoking status, marital status, resting heart rate, and blood pressure, revealed no significant differences between the e-waste workers and controls (two-sided *t*-test). However, the proportion of individuals reporting high stress levels was 12% higher among e-waste workers compared to controls. The study also classified e-waste workers into eight distinct job categories based on their specific roles in e-waste processing. Notably, the top four job categories comprised 82% of all e-waste workers, with wire burning being the most prevalent, accounting for 44% of the workers.

**Figure 1:**
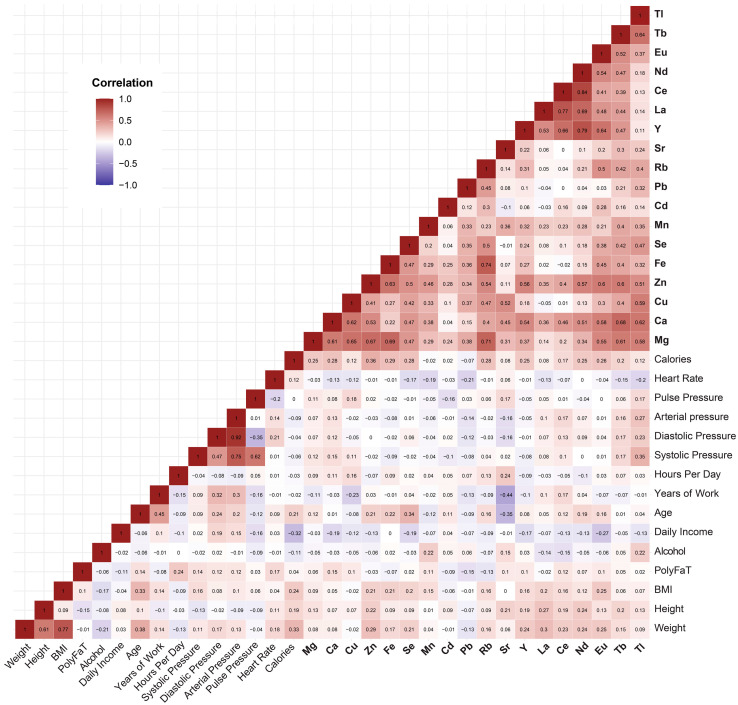
Correlation patterns between various study factors, with a particular emphasis on the relationships between metal concentrations and other participant characteristics. BMI: body mass index. Metal elements are highlighted in bold. Se, selenium; Tb, terbium; Cu, copper; Mg, magnesium; Ca, calcium; Sr, strontium; Rb, rubidium; Tl, thallium; Zn, zinc; Mn, manganese; Cd, cadmium; Fe, iron; La, lanthanum; Ce, cerium; Eu, europium; Nd, neodymium; Pb, lead; Y, yttrium.

A correlation analysis was conducted between the concentrations of 18 metals in the blood along with 15 other natural characteristics of the subjects. The results revealed that the metals were predominantly positively correlated with each other but showed no significant correlations with the other descriptors (Figure 1). This indicates that multiple metals accumulate significantly in the blood in e-waste workers, independent of their natural traits, further emphasizing the distinct impact of e-waste exposure on metal accumulation in the body.

### 3.2. Exposomics Data Analysis Workflow

**Figure 2:**
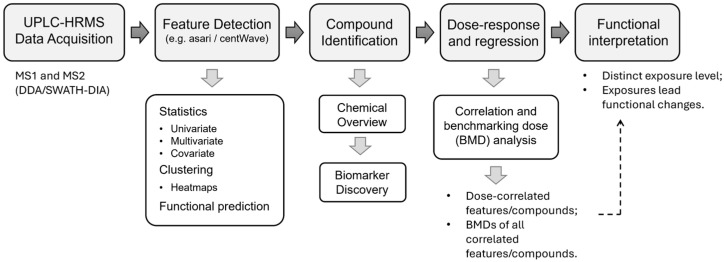
Workflow for exposomics data analysis implemented in this study.

The data analysis workflow was developed using the MetaboAnalystR 4.0 platform [31]. This workflow encompasses raw spectral processing, compound identification, correlation analysis with exposure data, and functional interpretation of the effects of exposures (Figure 2). Compound identification for both the metabolome and exposome was based on two pre-defined chemical databases (see Section 2).

### 3.3. Spectra Processing and Sensitivity Evaluation

Raw spectral data (MS1 level) were processed using two well-established feature detection algorithms, *asari* [22] and *centWave* [18]. Both algorithms detected > 14,000 features for all four modes (positive and negative modes in the C18 and HILIC columns, respectively). Principal component analysis (PCA) showed a clear separation between e-waste workers and controls (Supplementary Figures S1–S4), indicating significant perturbations in both the metabolome and exposome of e-waste workers compared to controls, underscoring the broad impact of e-waste exposure on biological systems.

**Figure 3:**
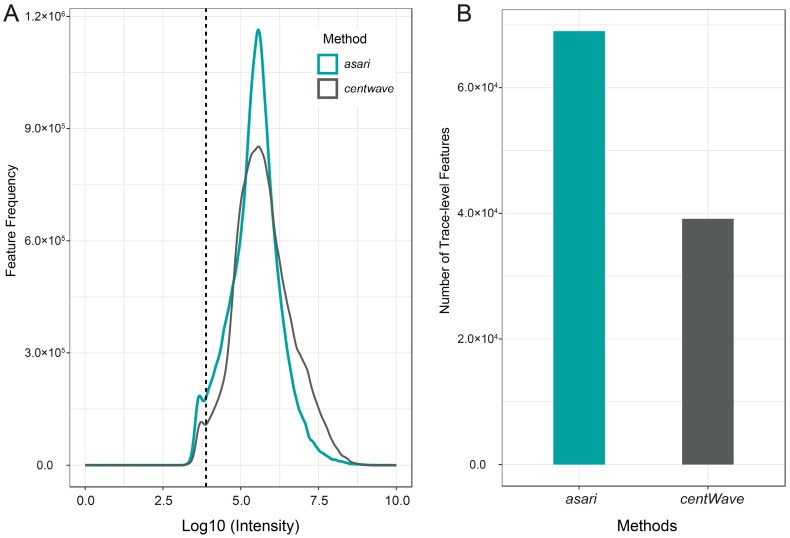
Sensitivity evaluation of feature detection for *asari* and *centWave*. (**A**) Distribution of all MS1 features in the C18 ESI^−^ mode detected by *asari* and *centWave* at different intensity levels. (**B**) A comparison of trace-level features (intensity < 10,000) detected by *asari* and *centWave*.

The sensitivity of feature detection was evaluated for the two algorithms. Unlike *centWave*, *asari* generates two datasets of results: the full feature table including all features detected by the algorithm, providing broad spectral coverage but with variable feature quality, and the preferred feature table containing only high-quality features. The latter was used for comparison with the results obtained by *centWave*. As illustrated in Figure 3A,B, *asari* consistently detected a greater number of features compared to *centWave*.

Trace-level features, defined as those with an intensity of less than 10,000, were particularly well-detected by *asari*, which identified > 76% more of these features in the C18 ESI^−^ mode than *centWave*. Similar patterns were also observed in the C18 ESI^+^ mode. However, no significant differences were observed between the two algorithms in the HILIC datasets (see Supplementary Figures S5 and S6). When comparing using the full feature table, *asari* demonstrated significantly higher sensitivity across all intensity levels, outperforming *centWave* in detecting a broader range of MS features (see Supplementary Figures S7–S10). These results highlight *asari*’s superior ability to detect trace-level and low-intensity features, particularly in the reverse-phase modes, making it a promising choice for exposomic analysis.

### 3.4. Feature Detection and Compound Identification

**Figure 4:**
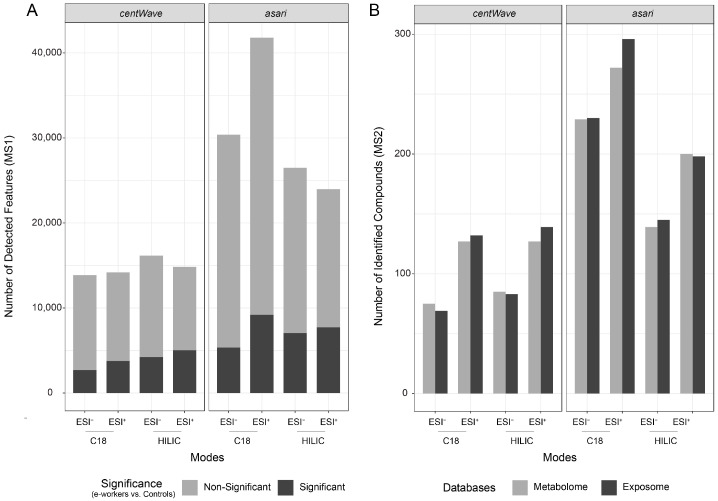
Summary of statistical analysis and compound identification results. (**A**) Statistical analysis of MS1 features across different modes using the *centWave* and *asari* algorithms. (**B**) Compound identification results from different chemical universes in various modes, based on MS1 features obtained from either *centWave* or *asari*. The modes include C18 ESI^−^, C18 ESI^+^, HILIC ESI^−^, and HILIC ESI^+^. Statistical significance was determined using a two-sided t-test (*p*-values adjusted by false discovery ratio (FDR), setting the threshold as 0.05).

As shown in Figure 4A, >14,000 MS1 features were detected by *centWave* across different analytical modes. Among them, 26.6% and 19.5% of features in the C18 ESI^+^ and C18 ESI^−^ modes, respectively, were identified as significantly altered in e-waste workers compared to controls. Additionally, more than 26% of the detected features were also found to differ significantly between the two groups in HILIC modes. In comparison, the *asari* algorithm detected > 23,000 features, representing a 64% increase in feature detection compared to *centWave*. Of these, more than 17% of features from the C18 ESI^+^ and C18 ESI^−^ modes were reported as significant, while over 26% of features from the HILIC modes were found to be significantly different in e-waste workers compared to controls. The statistics of all features across different modes are summarized in Supplementary Table S2.

MS2-based compound identification was performed using the complete MS1 feature list as the detection target. Candidates with a matching score greater than 80 (out of 100) were considered confidently identified. As shown in Figure 4B and Figure S11, the total number of compounds identified based on MS1 features detected by *asari* was significantly higher than those identified by *centWave* across different modes. The number of compounds identified from different chemical databases was generally comparable, with the exposome database yielding 2–4% more compounds than the metabolome database. As expected, *asari* shows a higher sensitivity in detecting features in the C18 modes compared to HILIC, leading to the identification of a greater number of compounds in these modes. In contrast, *centWave* exhibited a more balanced detection of MS1 features across the different modes, detecting similar numbers in both the C18 and HILIC datasets.

### 3.5. Overview of Exposome

**Figure 5:**
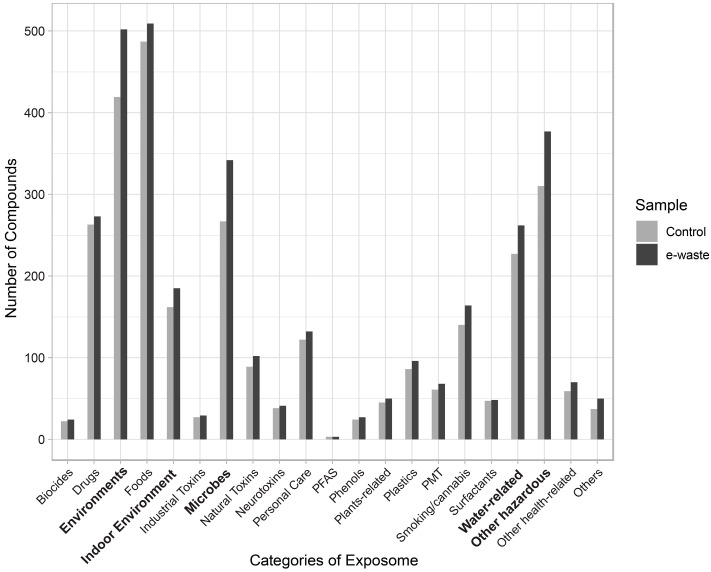
Summary of exposome categories identified in control and e-waste workers. PFAS, per- and polyfluoroalkyl substances. PMT, persistent, mobile, and toxic substances.

All of the chemicals from the exposome database were classified into >20 different exposure types (see Methods). Identified compounds from various analytical modes were then mapped to these exposure categories. Each compound was assigned to a specific exposure type such as environmental exposure, food, microbes, drinking water, pharmaceuticals, and other hazardous substances (Figure 5). We further compared the identified compounds between e-waste workers and controls. Across all exposure categories, e-waste workers exhibited a higher number of identified compounds. Notably, there was a significant increase in compounds related to environmental exposure, indoor environmental exposure, microbial exposure, water-related exposure, and hazardous chemicals in e-waste workers compared to controls. For example, CMPF (3-carboxy-4-methyl-5-propyl-2-furanpropionic acid), a uremic toxin associated with environmental and water-related exposure [43], was significantly elevated in e-waste workers. Additionally, two microbially sourced compounds, 2-ketobutyric acid and 9-decenoylcarnitine, were identified in e-waste workers [44,45]. Mirror plots illustrating the spectral matching patterns against reference libraries for these three compounds are presented in Supplementary Figure S12. Other categories showed similar levels of compound identification between the two groups.

### 3.6. Association Analysis with Heavy Metals

**Figure 6:**
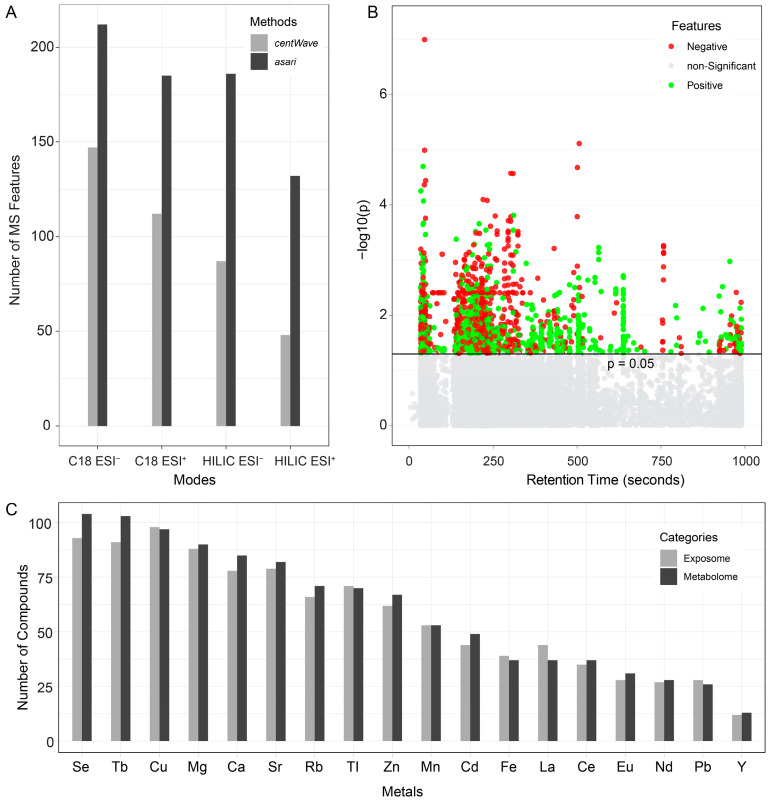
Correlation analysis results. (**A**) Summary of Pearson’s correlation analysis between metal concentrations and MS features detected by the *asari* and *centWave* algorithms across different analytical modes. (**B**) Linear regression analysis of MS features in the C18 ESI^−^ mode and their associations with copper (Cu) concentration groups. “Non-significant” refers to MS features with no significant associations. “Positive” indicates features showing a significant positive association with metal concentration, while “Negative” refers to features with a significant inverse association. (**C**) Summary of compounds from both metabolome and exposome chemical databases that were found to be significantly correlated with various metals. Se, selenium; Tb, terbium; Cu, copper; Mg, magnesium; Ca, calcium; Sr, strontium; Rb, rubidium; Tl, thallium; Zn, zinc; Mn, manganese; Cd, cadmium; Fe, iron; La, lanthanum; Ce, cerium; Eu, europium; Nd, neodymium; Pb, lead; Y, yttrium.

All MS features detected by both the *asari* and *centWave* algorithms were included in Pearson’s correlation analysis with blood metal concentrations. As shown in Figure 6A, the C18 ESI^−^ mode detected the highest number of MS features correlated to specific metals, while the HILIC ESI^+^ mode detected the fewest. Across all modes, *asari* identified more metal-correlated features than *centWave*, which is consistent with the overall higher number of features detected by *asari*.

From the perspective of individual metals, all metals, except europium (Eu), were found to be correlated with at least one MS feature. Notably, lanthanum (La), selenium (Se), magnesium (Mg), and cadmium (Cd) showed significant quantitative correlations (R > 0.5; *p* < 0.001; false discovery ratio, FDR, <0.1) with >100 features across different modes (Supplementary Figure S13). This suggests that these metals may play a substantial role in contributing to the biological changes observed in e-waste workers.

To further investigate the associations between metals and MS features, linear regression was performed for all metals and MS features across different analytical modes. Several metadata factors, including age, BMI, stress levels, and years of work, were included as covariates to control for potential confounders. The results revealed that between 700 and 5000 features were significantly correlated with metal concentrations, depending on the specific metal. Some MS features showed a consistent positive correlation with higher metal concentrations, while others were inversely correlated (Figure 6B, Supplementary Figures S14–S16). Additionally, we summarized the number of identified compounds that are significantly correlated with the concentrations of various metals. As shown in Figure 6C, all 18 metals were significantly associated with 12~110 compounds from the metabolome and exposome libraries. Among these, selenium (Se), terbium (Tb), and copper (Cu) were the top three metals associated with the highest number of compounds. Detailed information on these compounds is provided in Supplementary Tables S3 and S4. These correlations between MS features and metal concentrations suggest that varying levels of metal exposure in e-waste workers may potentially impact diverse biological functions.

### 3.7. Functional Analysis

**Figure 7:**
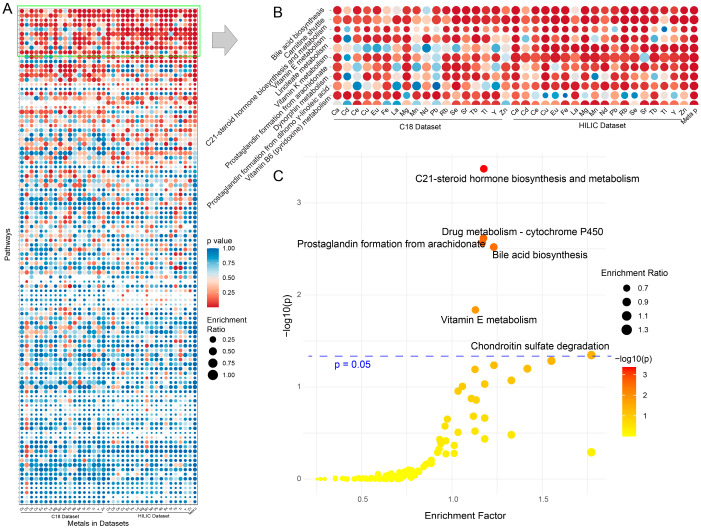
Results from functional enrichment analysis. (**A**) The bubble plot summarizing all functional enrichment results across all metals. Pathways are sorted by *p*-values, with the most significant pathways (lowest *p*-values) listed at the top. (**B**) The top 10 pathways from panel (**A**) representing the most significantly enriched pathways based on the comparison of metal levels. (**C**) Scatter plot of functional enrichment analysis from the C18 dataset, comparing e-waste workers to controls. The enrichment factor of a pathway is the ratio of significant pathway hits in the user-uploaded data to the expected number of hits for that pathway. All analyses were performed based on the MS features detected using the *asari* algorithm.

To investigate the biological perturbations associated with varying levels of blood metals, *mummichog* [46] was employed to explore functional changes linked to MS features that are significantly associated with metal concentrations across both C18 and HILIC datasets as well as those that differed between e-waste workers and controls. The results from the enrichment analysis were integrated at the pathway level, and all perturbed pathways related to metal concentration changes were summarized in a bubble plot (Figure 7A,B, Supplementary Table S5). Notably, the most significantly perturbed pathways were consistently reported across all metals and datasets. The analysis focused on the top 10 pathways (integrated *p* with Fisher’s method [47]), which are related to bile acid biosynthesis, C21-steroid hormone biosynthesis and metabolism, prostaglandin formation, as well as the metabolism of the carnitine shuttle, linoleate, vitamins, and dynorphin. Drug metabolism of CYP450 was found to be significantly altered in e-waste workers compared to controls based on the C18 dataset (Figure 7C). Chondroitin sulfate degradation shows a marginally significant *p*-value (*p* = 0.05). No more distinct pathways were observed in the HILIC dataset (Supplementary Figure S17). These findings highlight the widespread metabolic disruptions in e-waste workers, particularly in pathways critical to hormone regulation, energy metabolism, and detoxification processes.

### 3.8. Dose–Response Analysis

Dose–response analysis is a method used to assess the relationship between the dose of a substance, such as a drug, toxin, metal, or other chemical exposures, and the associated biological responses. It aims to estimate safe exposure levels and the concentration at which a substance has half its maximal effect [48]. This approach is widely used in toxicology [49] and pharmacology studies [50]. Herein, all MS features meeting significance criteria in linear regression analyses were included in a dose–response evaluation. Metal concentrations were treated as the input dose, while MS feature intensities served as the response variables (see Section 2). The half-maximal effective concentrations (EC50) were utilized as the benchmark doses (BMDs) for each metal concerning its corresponding MS feature. We tested all features showing significant linear relationships, with over 1700 MS features across different modes demonstrating a significant fit to the dose–response model for all metals (*p* < 0.05), encompassing 38–72% of the linear-regressed MS features (Supplementary Table S6). The remaining features either did not converge or failed to fit within the model (*p* > 0.05).

**Figure 8:**
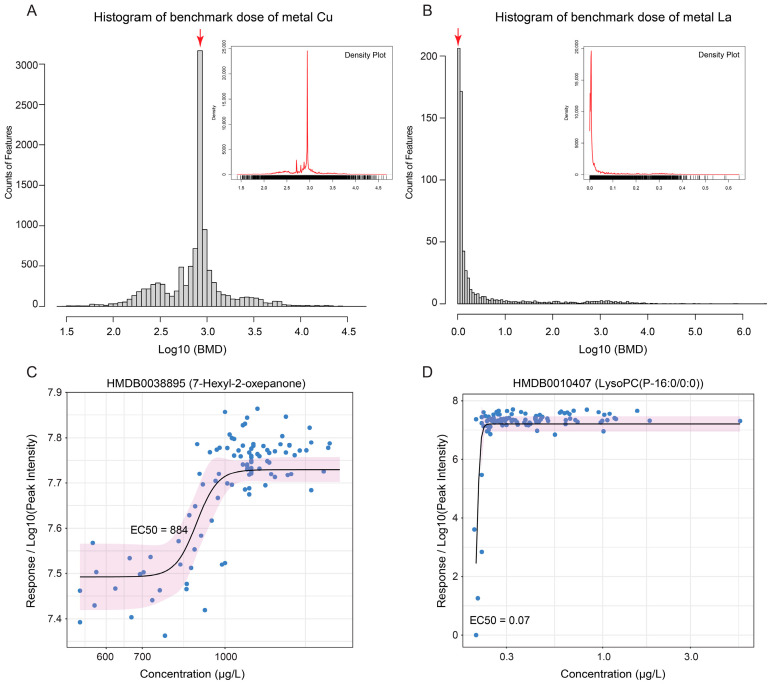
Dose–response analysis results. (**A**) Distribution of benchmark doses (BMDs) for the metal copper (Cu). The BMDs of MS features associated with Cu exhibit a normal distribution, with most BMDs concentrated within the Log10 (BMD) range of 2.90–2.95. (**B**) Distribution of benchmark doses (BMDs) for the metal lanthanum (La). In contrast, the BMDs of MS features associated with La displayed a significantly skewed distribution, with most BMD values falling within the initial range of 0–0.1 on the Log10 (BMD) scale (first two histogram intervals). Red arrows, Log10 (BMD) intervals with the most MS features. The dose–response curves for 7-Hexyl-2-oxepanone (**C**) and LysoPC (P-16:0/0:0) (**D**) illustrate distinct dose–response trends for Cu and La, respectively, highlighting the differences in MS feature response patterns to each metal.

Summaries of EC50 values (BMDs) for each metal were displayed using histogram and density plots (Figure 8A,B). Notably, two distinct distribution patterns emerged. Metals such as magnesium (Mg), calcium (Ca), zinc (Zn), copper (Cu), iron (Fe), lead (Pb), rubidium (Rb), manganese (Mn), strontium (Sr), and selenium (Se) exhibited a normal distribution of EC50 values, suggesting that effective doses correlate with the accumulation of these metals (Figure 8A and Figures S18–S26). Conversely, other metals, including cadmium (Cd), cerium (Ce), europium (Eu), lanthanum (La), neodymium (Nd), terbium (Tb), thallium (Tl), and yttrium (Y), displayed significantly skewed or nearly exponential EC50 distributions (Figure 8B and Figures S27–S33), indicating an extremely low EC50 threshold. In these cases, we identified two compounds, 7-Hexyl-2-oxepanone and LysoPC (P-16:0/0:0), each showing a strong, significant dose–response relationship with Cu and La, respectively (*p* < 1 × 10^−5^ and correlation R > 0.8) (Figure 8C,D).

## 4. Discussion

Humans are exposed to countless chemicals throughout their lifetimes, with many of these exposures being environmental or occupational in nature [51,52]. In this study, we collected 100 whole blood samples from e-waste workers and utilized an LC-MS/MS platform to detect all MS features. A comprehensive workflow integrated with curated metabolome and exposome reference libraries was designed for data processing, analysis, and interpretation. This study not only confirmed the effectiveness of the established workflow but also uncovered distinct metabolic patterns, metal-associated MS features, and significant functional disruptions in e-waste workers.

Over the past two years, several workflows and concepts have been developed for exposomics data processing, including frameworks such as rexposome and ExWAS [52,53,54,55,56]. However, none of these existing workflows have integrated key stages of data analysis—from raw spectral processing and statistical analysis to feature annotation, compound identification, and functional interpretation. Unlike metabolomics, which primarily focuses on endogenous features, exposomics emphasizes the detection of MS features related to environmental exposures and exogenous chemicals [37,57]. These exogenous features are typically present at trace levels, making them challenging to detect using traditional metabolomics feature detection methods, such as *centWave*. *centWave* filters peaks based on a wavelet transform model [18], which tends to introduce bias toward peaks with well-defined Gaussian shapes. In contrast, *asari*, a newer detection algorithm, identifies features by aggregating peak signals across samples, making it more sensitive to features of low intensity or with non-Gaussian shapes [22]. This enhanced sensitivity makes *asari* particularly well-suited for detecting exposomics features. Our study highlights the better performance of *asari* in detecting features across multiple modes, although *centWave* also exhibited similar trends. Furthermore, this study demonstrates the value of using multiple analytical modes and algorithms, as this approach allows for a more comprehensive assessment of both metabolomic and exposomic profiles. By employing diverse methods, we increase the likelihood of identifying biologically relevant compounds to better understand risks of the chemical exposures impacting e-waste workers.

The health of e-waste workers is severely compromised due to environmental contamination, primarily through the inhalation or ingestion of polluted air, water, and food [58]. In this study, we observed significant differences in the metabolic and exposomic profiles of e-waste workers compared to controls, aligning with the previous reports [59,60]. The results clearly show the different exposomic profiles between e-waste workers and controls. The significantly distinct chemical categories point to exposures from the environment, drinking water, hazardous chemicals, and microbes. These factors are directly related to the occupational characteristics of e-waste workers. Microbial exposures also contribute to the perturbation of the exposome of e-waste workers. This may be associated with host–microbiota interactions. The high proportion of significantly altered features across multiple detection modes underscores the extensive biological impact of e-waste exposure on various metabolic pathways.

Several functional pathways were identified as significantly perturbed in e-waste workers, including those involved in bile acid biosynthesis, C21-steroid hormone biosynthesis and metabolism, drug metabolism, prostaglandin formation, linoleate metabolism, the carnitine shuttle, and vitamin K metabolism. Among these, previous studies have already established associations between hormonal imbalances, liver function disruptions, and prostaglandin biosynthesis in e-waste workers [61,62,63]. Drug metabolism and bile acid biosynthesis are directly linked to liver functionality, further emphasizing the impact of e-waste exposure on hepatic health. Additionally, disruptions in linoleate metabolism and the carnitine shuttle have been previously correlated with metal exposure [64] or volatile organic compounds [65], respectively. Vitamin E pathways have been demonstrated to play a protective role against the toxicity of heavy metals [66,67,68] and organophosphorus chemicals [69]. Notably, our study is the first to report dysfunction in vitamin K metabolism and chondroitin sulfate degradation among e-waste workers, a novel finding that warrants further investigation. This study emphasizes the broad range of biological systems affected by e-waste exposure and the need for continued investigation into the long-term health consequences for e-waste workers. The detailed molecular mechanisms leading to this disruption remain unclear and require additional elucidation in future research.

Metal exposure is a common occupational hazard for e-waste workers [70]. In this study, we observed strong correlations and clustering among multiple metals, which is expected given that these metals may originate from direct or indirect occupational exposures. We evaluated the associations between MS features and metal levels, identifying hundreds of thousands of features. This study provides a comprehensive overview of how varying metal concentrations correlate with MS features, offering valuable insights into the metabolic and exposomic changes induced by metal exposure.

To gain a deeper understanding of the quantitative effects of metals on the perturbation of both the metabolome and exposome, we conducted a dose–response analysis examining the concentrations and intensities of all linear-regressed features. Our results revealed that thousands of MS features exhibited significant fitting to a dose-response curve. Notably, we identified two distinct types of distributions for BMDs: normal distribution and significantly skewed distribution. The normal distribution was primarily associated with essential micronutrient metals critical for human health, such as magnesium (Mg), calcium (Ca), zinc (Zn), copper (Cu), iron (Fe), manganese (Mn), strontium (Sr), and selenium (Se) [71]. Additionally, it included metals that exhibit tolerance at low doses in humans, such as lead (Pb), as well as metals with inherently low toxicity, such as rubidium (Rb). In contrast, other metals displayed significantly skewed distribution, including cadmium (Cd), cerium (Ce), europium (Eu), lanthanum (La), neodymium (Nd), terbium (Tb), thallium (Tl), and yttrium (Y). These metals are classified as heavy metals [71], typically associated with low to medium toxicity or radioactivity [72,73]. The presence of these heavy metals at extremely low doses in the bloodstream can lead to significant alterations in metabolic profiles. This analysis highlights the heterogeneous nature of dose–response relationships among various metals, particularly in the context of e-waste workers.

One of the main focuses of this study is the association between altered metal concentrations and biological functions. Using linear regression with adjustment to several common covariates, we have identified multiple perturbed pathways that are consistently reported as significantly altered across different metals, which is expected given the strong correlations among these metals. Several of the pathways that were disrupted between e-waste workers and controls were also consistently linked to changes in metal levels, highlighting the crucial role of metal exposure in driving biological alterations in e-waste workers.

Many other pathways were also significantly associated with changes in metal levels. For instance, vitamin D3 metabolism and dynorphin metabolism are two pathways that exhibited considerable changes across most groups of metals. Previous studies have demonstrated a link between vitamin D3 metabolism and several metals, such as calcium [74,75] and lead [75,76,77]. Dynorphin, a class of opioid neuropeptides involved in pain, addiction, and mood regulation, has been reported to undergo metabolic dysfunction following exogenous exposure in animal models, potentially contributing to the onset of neurodegenerative diseases [78]. Overall, the functional perturbations observed in this study underscore the remarkable influence of metal exposure on biological processes in e-waste workers. This highlights the critical need to further investigate the long-term health consequences of such exposures.

Compared to traditional metabolomics studies, where serum or plasma is typically used, this study employed whole blood samples from participants for LC-MS/MS-based feature detection. This approach allows for the inclusion of a more comprehensive set of small molecules [31], particularly trace-level features that might otherwise be missed. However, there are several limitations to our study. First, we did not quantify certain metals such as gold and silver, which are known to be common exposures among e-waste workers [79]. The absence of these metals could limit the scope of our findings. Second, compound identification in this study was based on MS2 spectra, which, while informative, requires further confirmation with standard references in follow-up studies. Third, the cross-sectional nature of our study presents another limitation. Longitudinal studies based on cell lines or animal models would be necessary to elucidate the detailed molecular mechanisms by which metal exposure affects the health of e-waste workers. Finally, while whole blood samples provide the most complete set of small molecules in human biofluids, urine may be more suitable for assessing the broader metabolic status and more feasible for biomarker discovery [80,81]. Future studies should consider integrating urine analysis to provide a more comprehensive view of metabolic perturbations. This will allow for a comprehensive understanding of the biological impact of metal exposures on e-waste workers.

## 5. Conclusions

E-waste processing presents a global concern, with e-waste workers facing prolonged exposure to toxic metals and associated health risks. This study conducted a comprehensive untargeted metabolomics analysis using a newly developed exposomics data analysis workflow. Our findings demonstrate that the *asari* algorithm is more sensitive in terms of detecting a greater number of MS features, especially trace-level features, making it more suitable for exposomics research. Metal exposures were associated with significant changes in metabolic profiles, impacting multiple biological pathways involved in liver function, vitamin metabolism, and hormonal regulation. These findings contribute to a deeper understanding of the metabolic and functional perturbations associated with metal exposures and underscore the pressing need for further investigation into the health effects of e-waste processing on vulnerable populations.

## Figures and Tables

**Table 1:** Demographic characteristics of all subjects in this study.

Items	E-Waste Workers (n = 100)	Controls (n = 49)
BMI (kg/m^2^, mean ± SD)	21.76 ± 2.73	23.87 ± 3.47
Age (mean ± SD)	25.40 ± 6.33	32.48 ± 10.36
Marital Status (Married/Single, n)	55/45	20/29
Smoking (Yes/No, n)	27/73	6/43
Heart Rate (mean ± SD)	74.67 ± 12.37	76.12 ± 14.02
Systolic Blood Pressure (mmHg, mean ± SD)	123.57 ± 12.72	128.31 ± 18.21
Diastolic Blood Pressure (mmHg, mean ± SD)	73.98 ± 9.38	76.96 ± 12.48
Stress (Stressed/Non-stressed, n)	61/39	24/25
Jobs (n)	Burning wires only (44); Ash/wire collection after burning (12); Collecting e-waste (13); Sorting e-waste (13); Others * (18).	Non-e-waste workers

* Other jobs include repairing e-waste, removing covers of wires, buying or trading e-waste, and burning e-waste.

## Data Availability

The original data presented in the study are openly available from this link: https://drive.google.com/drive/folders/1b8_9A14dmXQpyy-FckBUTx1Jen2WKAUH (Access date: 30 October 2024).

## Supplementary Materials

The following supporting information can be downloaded at: https://www.mdpi.com/article/10.3390/metabo14120671/s1, Figures S1–S4: PCA score plot of C18 ESI^−^, C18 ESI^+^, HILIC ESI^−^, HILIC ESI^+^ modes, respectively. Figures S5 and S6: Sensitivity evaluation of feature detection for *asari* and *centWave* for HILIC ESI^−^ and HILIC ESI^+^ modes, respectively. Figures S7–S10: Sensitivity evaluation of feature detection for *asari* (full feature data) and *centWave* across different modes, C18 ESI^−^, C18 ESI^+^, HILIC ESI^−^ and HILIC ESI^+^, respectively. Figure S11: Summary of all identified compounds by either algorithms or modes. Figure S12: Mirror plots of MS/MS spectra of three identified compounds. Figure S13: Summary of MS features correlated with metals. Figure S14: Manhattan plots of metals, Ca, Cd, Ce, Cu, Eu and Fe in C18 ESI^−^ mode. Figure S15: Manhattan plots of metals, La, Mg, Mn, Nd, Pb and Rb in C18 ESI^−^ mode. Figure S16: Manhattan plots of metals, Se, Sr, Tb, Tl, Y and Zn in C18 ESI^−^ mode. Figure S17: Scatter plot of functional analysis based on HILIC dataset (e-waste workers vs. Control). Figures S18–S33: Histograms and density plots of all benchmark doses of metals, Mg, Ca, Zn, Fe, Pb, Rb, Mn, Sr, Se, Cd, Ce, Eu, Tb, Nd, Tl and Y, respectively. Supplementary Table S1: Statistics and summary of all detected MS features across different modes and methods. Supplementary Table S2: All parameters used for *centWave*. Supplementary Table S3: Linear regression analysis results (*p* values) of all metals and exposome compounds. Supplementary Table S4: Linear regression analysis results (*p* values) of all metals and metabolome compounds. Supplementary Table S5: Integrated functional analysis results across all metals. Supplementary Table S6: Summary of all MS features fitting dose-response curves.
