# Supplementary material for: Comprehensive Blood Metabolome and Exposome Analysis, Annotation, and Interpretation in E-Waste Workers
Source: Metabolites. 2024 Dec 2;14(12):671. doi: 10.3390/metabo14120671 (PMC11677618; doi:10.3390/metabo14120671)
Supplement: Supplementary file 1 [file metabolites-14-00671-s001.zip › Manuscript_supplementary_materials.pdf]

Supplementary Materials

Comprehensive Blood Metabolome and Exposome Analysis, Annotation, and Interpretation in E-Waste Workers

Zhiqiang Pang [1], Charles Viau [1], Julius N. Fobil [2,3], Niladri Basu [1] and Jianguo Xia [1,*]

[1] Faculty of Agricultural and Environmental Sciences, McGill University, Ste-Anne-de-Bellevue, QC H9X 3V9, Canada; zhiqiang.pang@mail.mcgill.ca (Z.P.)
[2] School of Public Health, University of Ghana, Legon, Accra P.O. Box LG 13, Ghana; jfobil@ug.edu.gh
[3] West Africa Center for Global Environmental & Occupational Health, College of Health Sciences, Legon, Accra P.O. Box LG 13, Ghana
* Correspondence: jeff.xia@mcgill.ca; Tel.: +1-514-398-8668

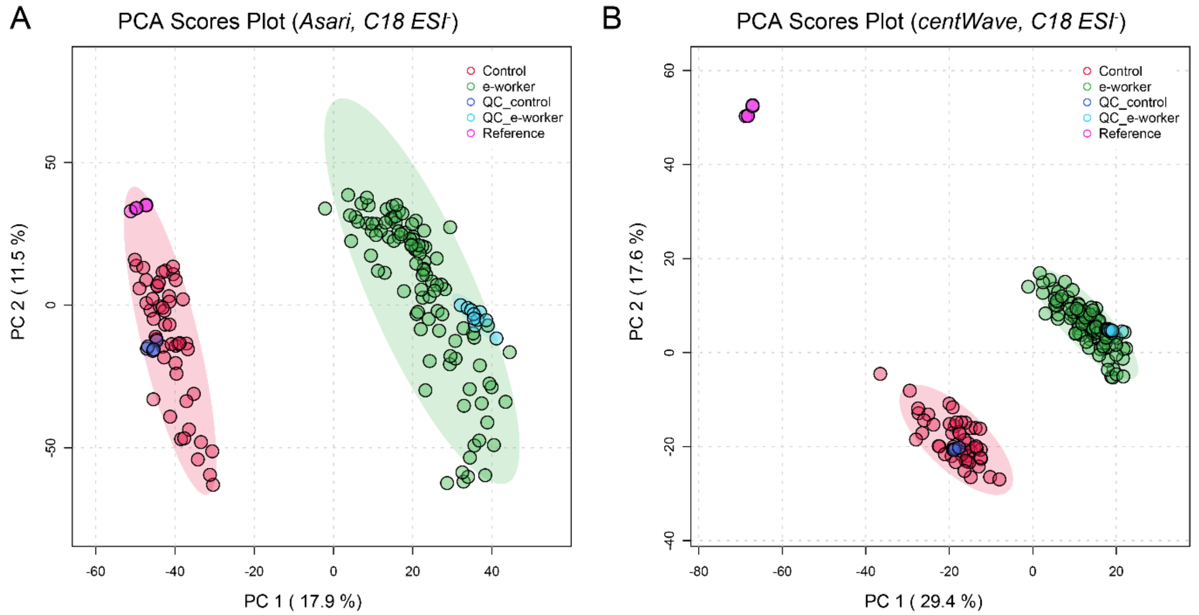

Figure S1. PCA score of C18 ESI$^-$ mode.

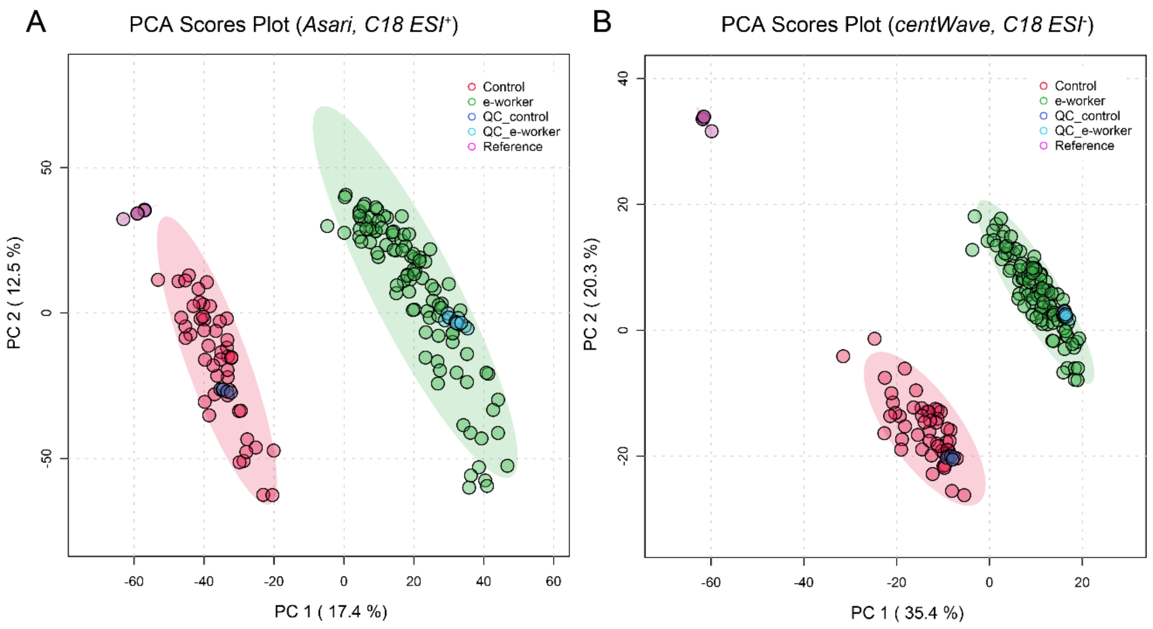

Figure S2. PCA score of C18 ESI^{+} mode.

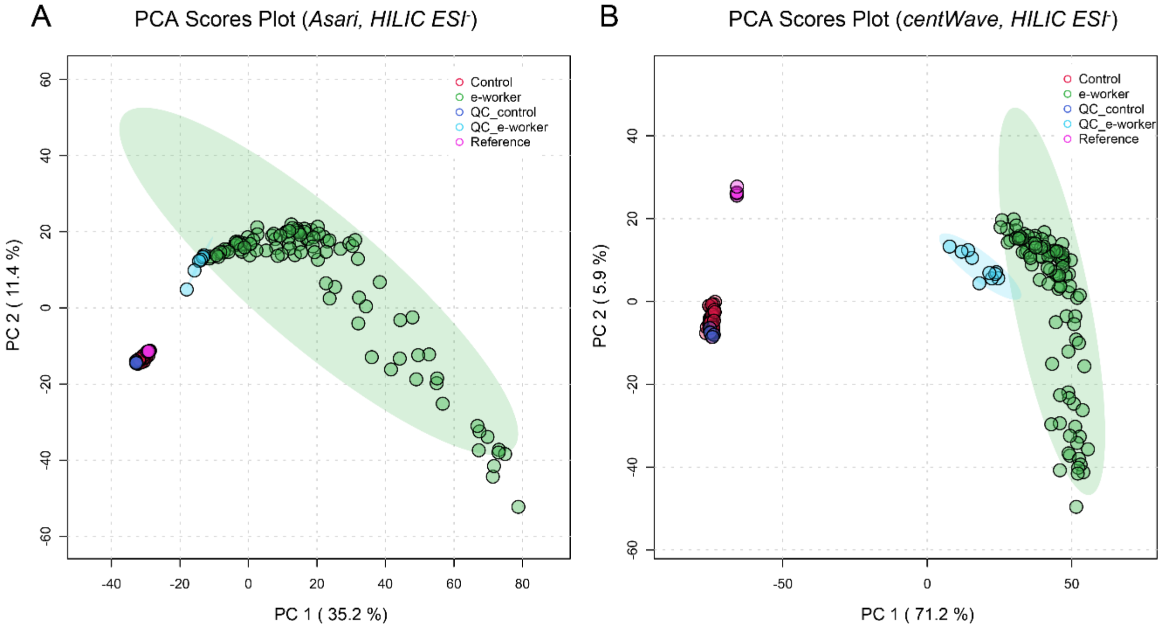

Figure S3. PCA score of HILIC ESI^{-} mode.

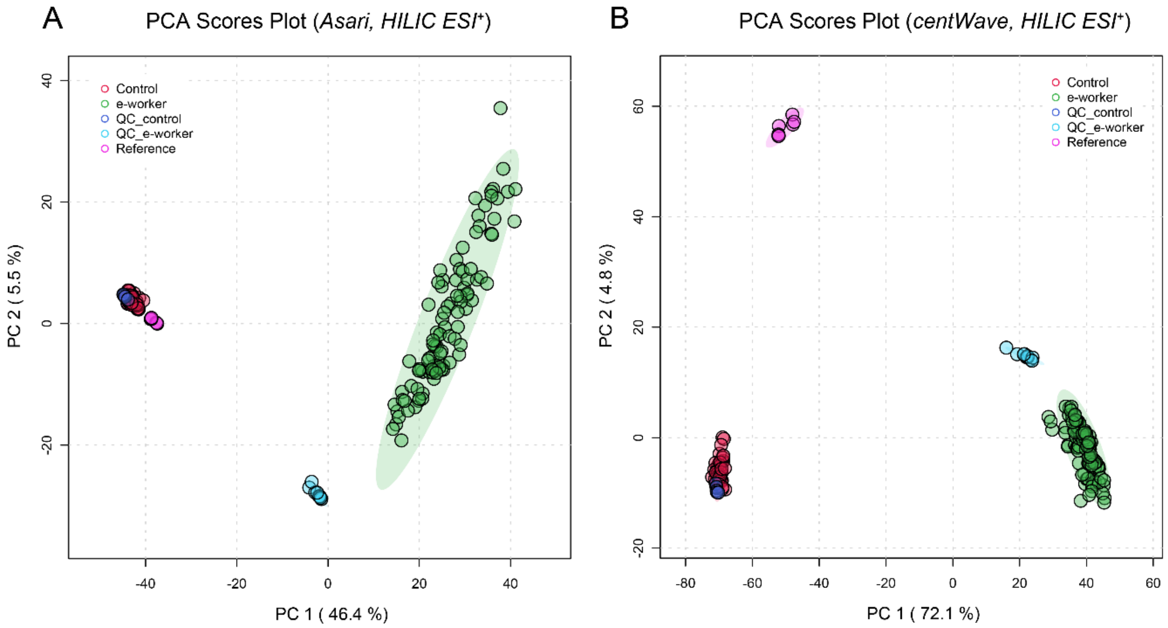

Figure S4. PCA score of HILIC ESI$^+$ mode.

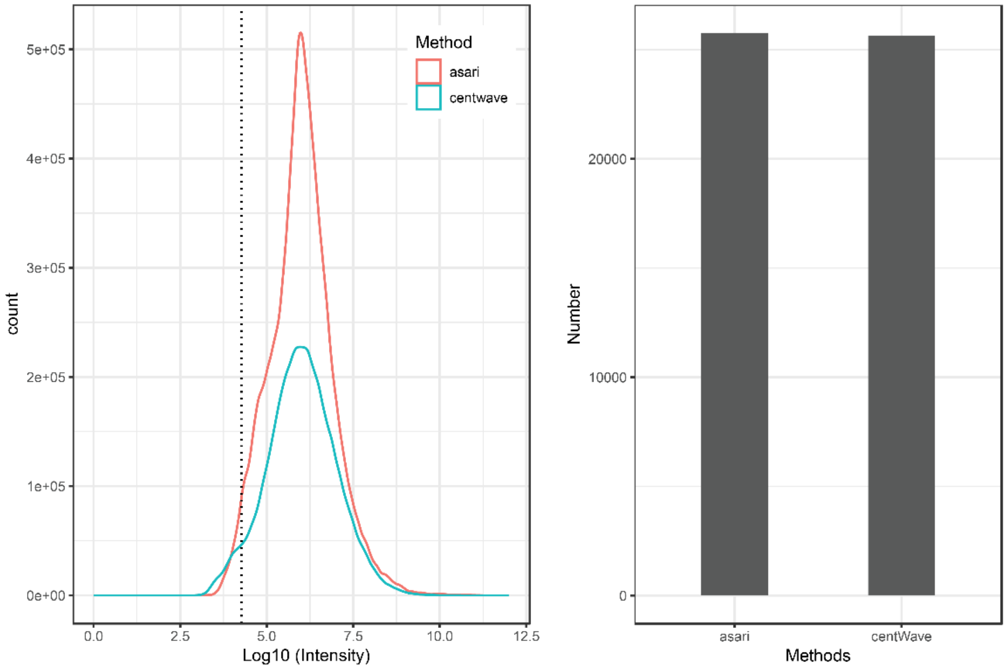

Figure S5. Sensitivity Evaluation of Feature Detection for *asari* and *centWave* (HILIC ESI$^-$).

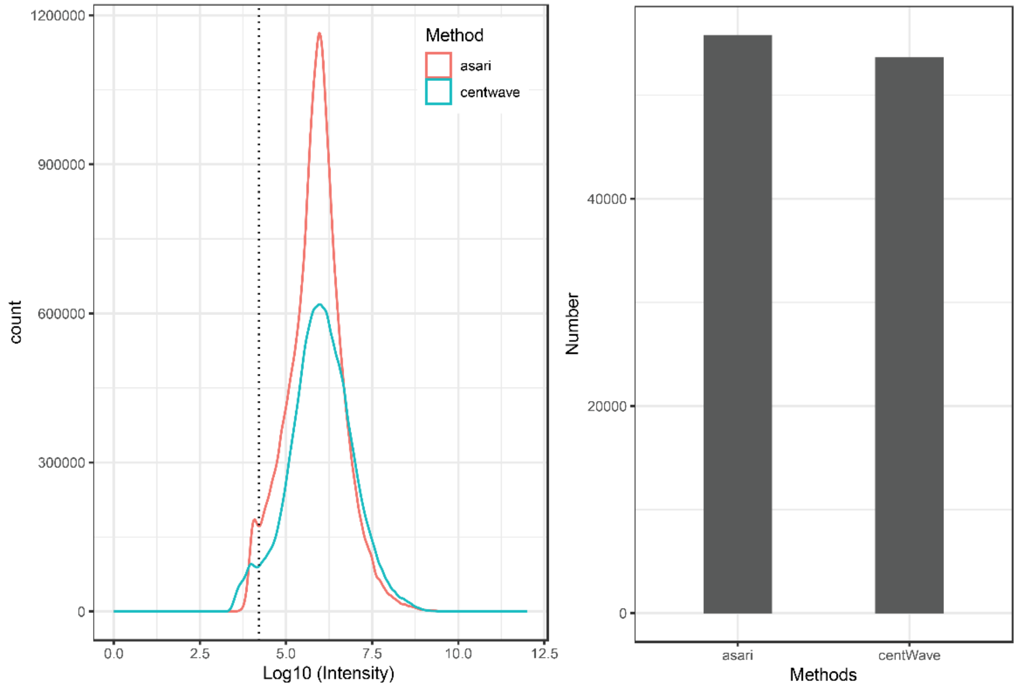

Figure S6. Sensitivity Evaluation of Feature Detection for *asari* and *centWave* (HILIC ESI$^+$).

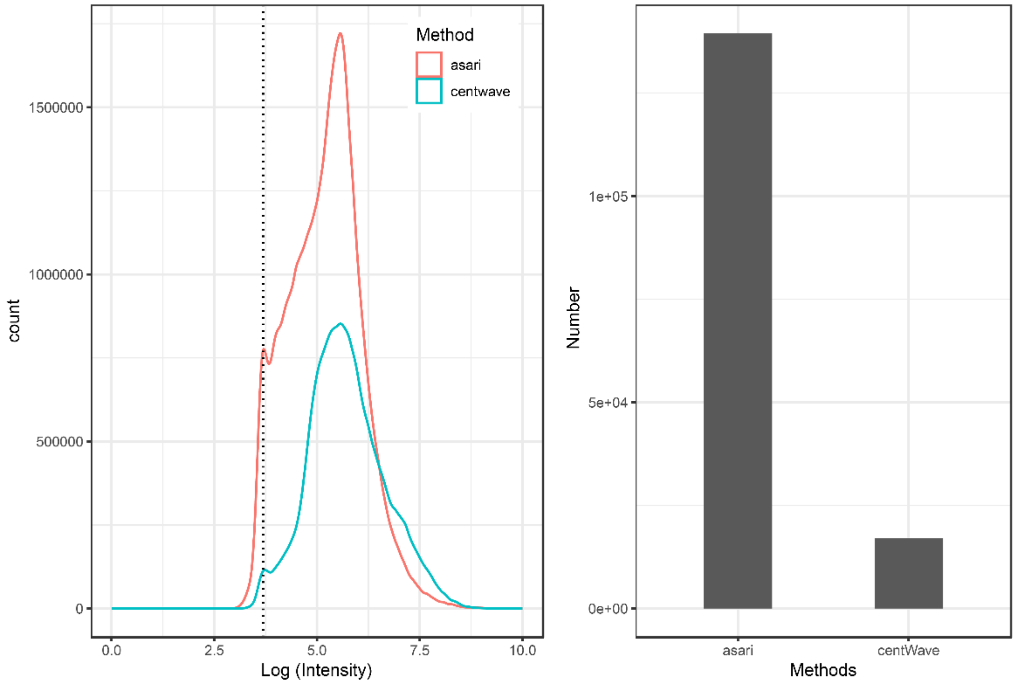

Figure S7. Sensitivity Evaluation of Feature Detection for *asari* (full feature data) and *centWave* (C18 ESI$^-$).

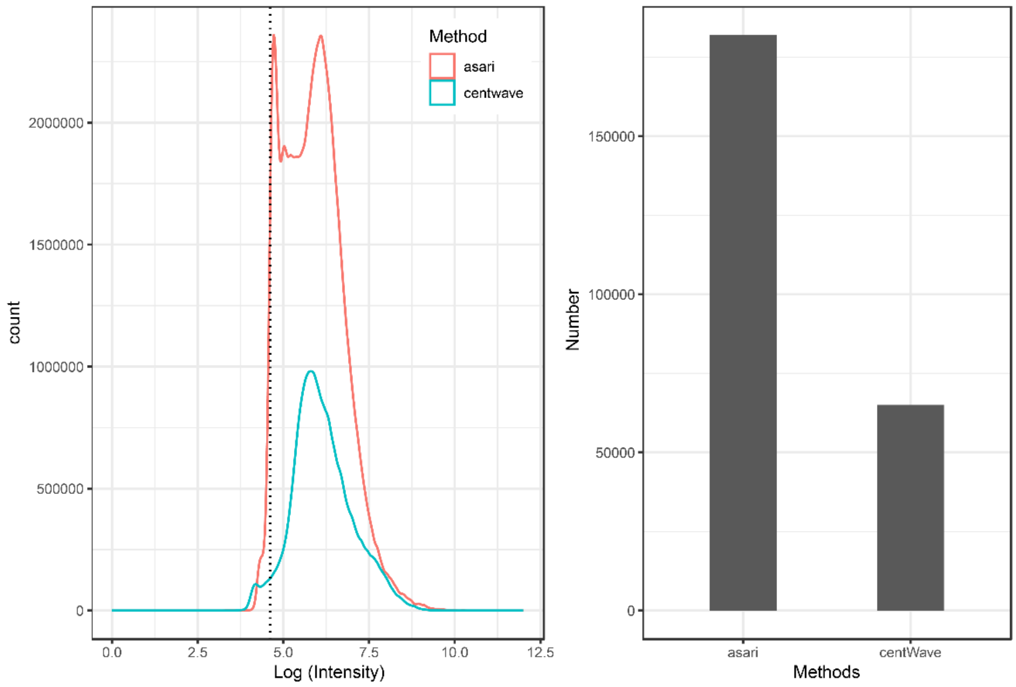

Figure S8. Sensitivity Evaluation of Feature Detection for *asari* (full feature data) and *centWave* (C18 ESI^{+}).

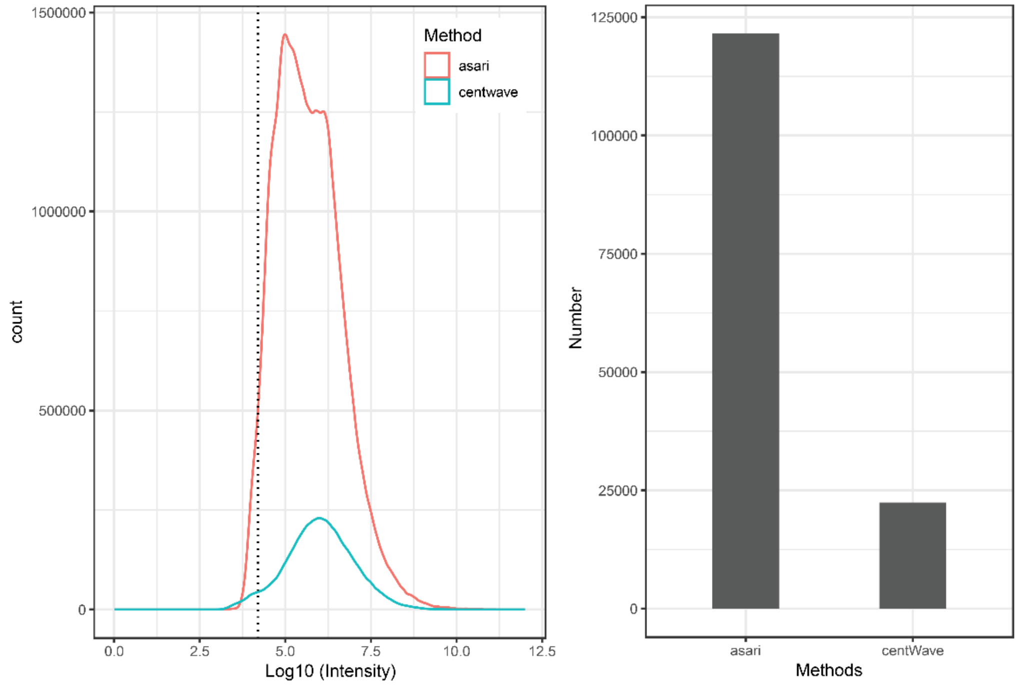

Figure S9. Sensitivity Evaluation of Feature Detection for *asari* (full feature data) and *centWave* (HILIC ESI^{-}).

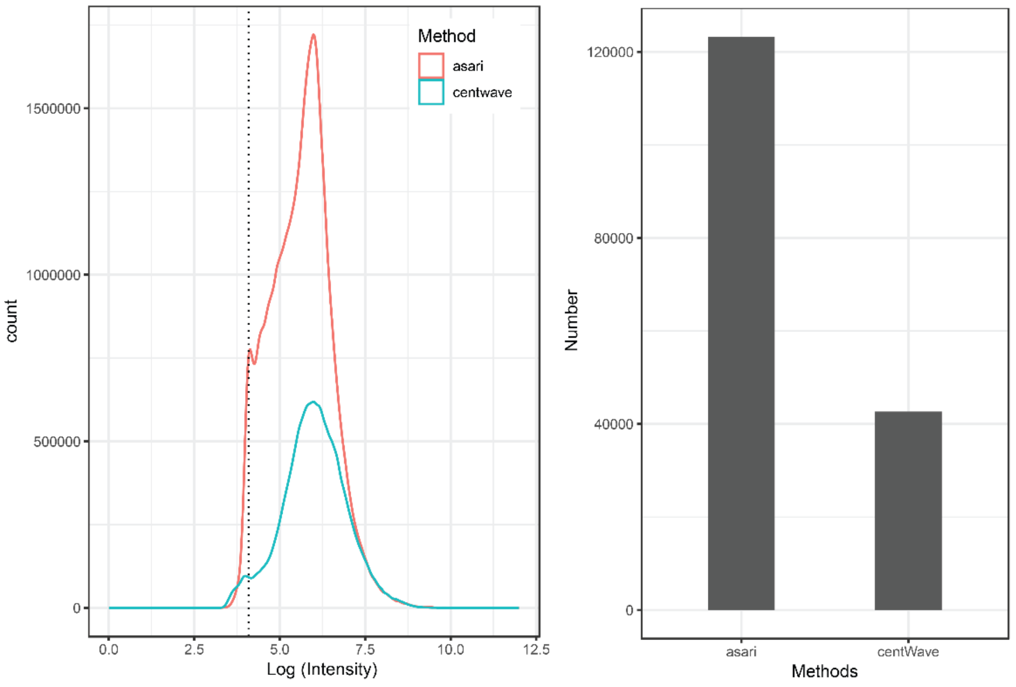

Figure S10. Sensitivity Evaluation of Feature Detection for *asari* (full feature data) and *centWave* (HILIC ESI$^+$).

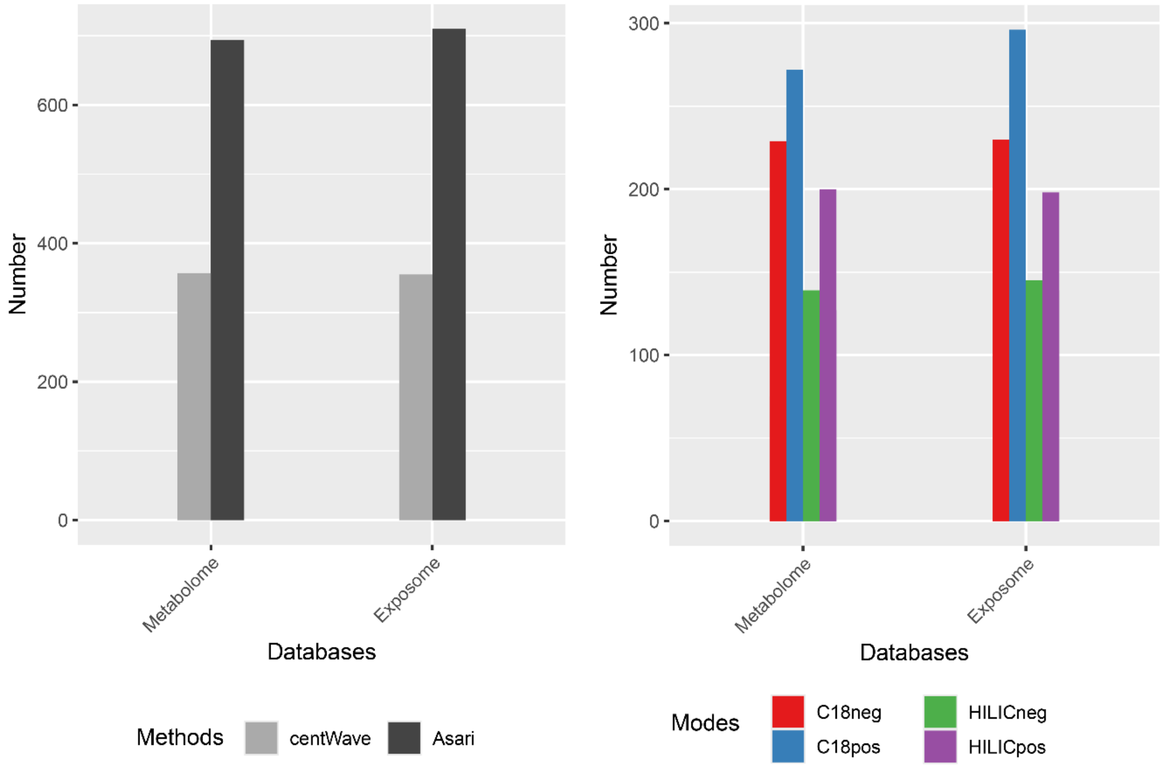

Figure S11. Summary of all identified compounds by either algorithms or modes.

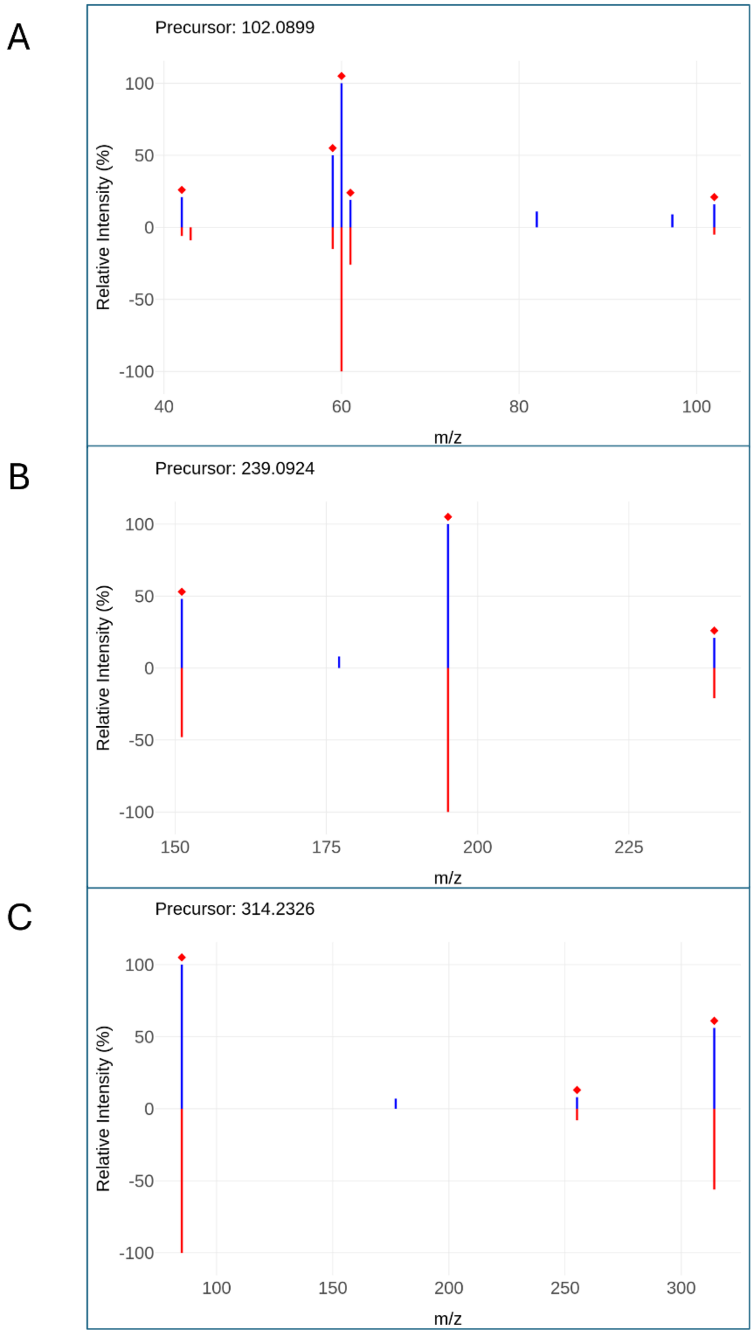

Figure S12. Mirror plots of MS/MS spectra of three identified compounds. (A) The mirror plot of 2-ketobutyric acid, which was identified based on the reference library from MoNA. (B) The mirror plot CMPF (3-carboxy-4-methyl-5-propyl-2-furanpropionic acid), which was identified based on the reference library from MoNA. (C). The mirror plot of 9-decenoylcarnitine, which

was identified based on the reference library from GNPS. The similarity score of all these three compounds are over 0.9.

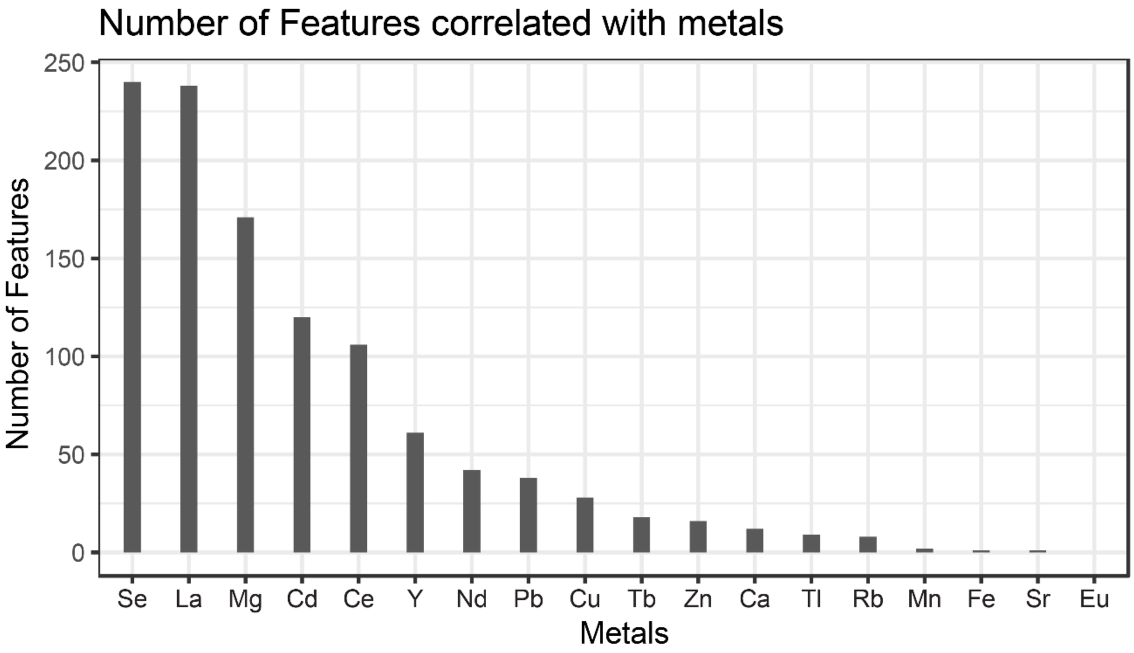

Figure S13. Summary of MS features correlated with metals.

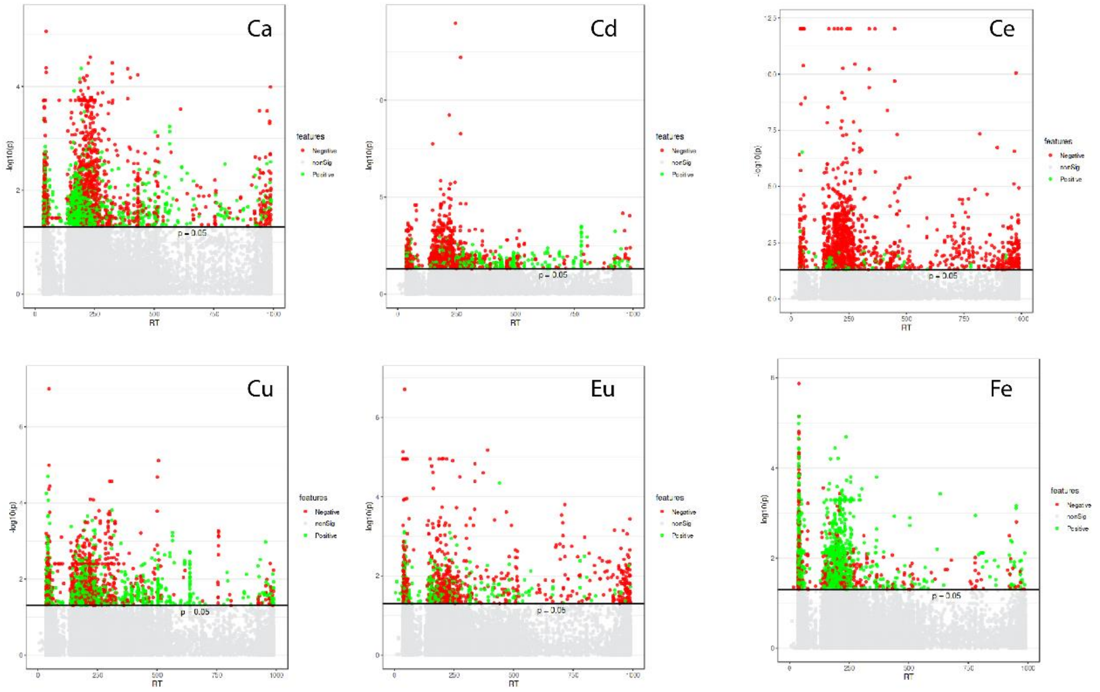

Figure S14. Manhattan plots of metals, Ca, Cd, Ce, Cu, Eu and Fe in C18 ESI^{-} mode.

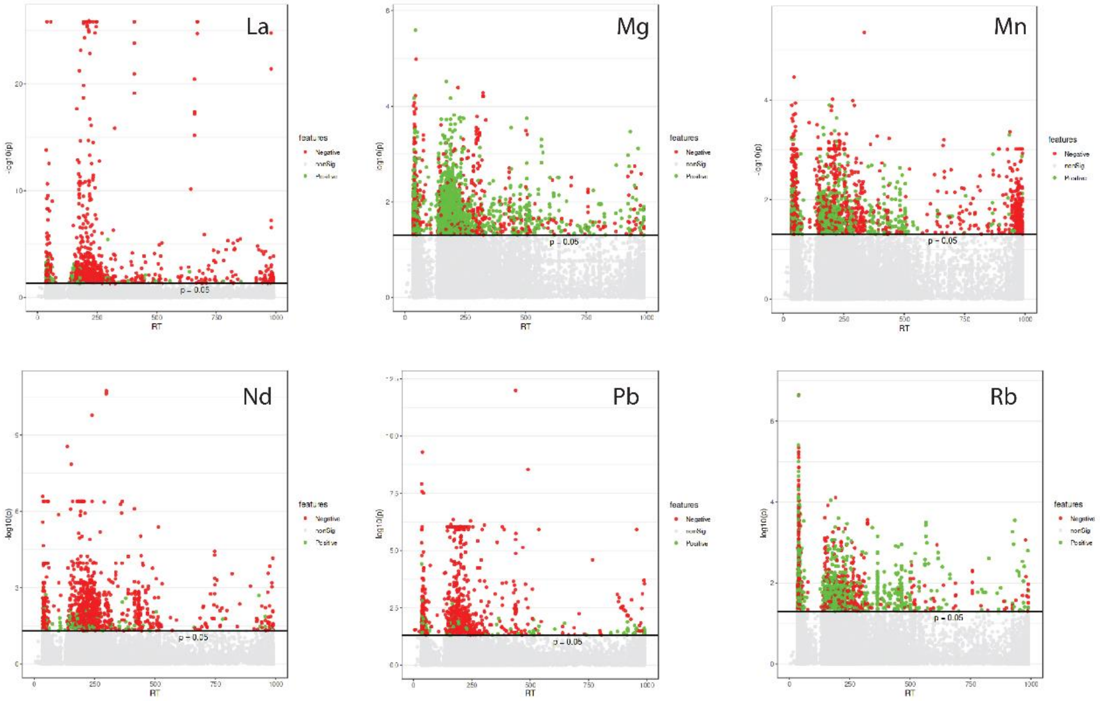

Figure S15. Manhattan plots of metals, La, Mg, Mn, Nd, Pb and Rb in C18 ESI^{-} mode.

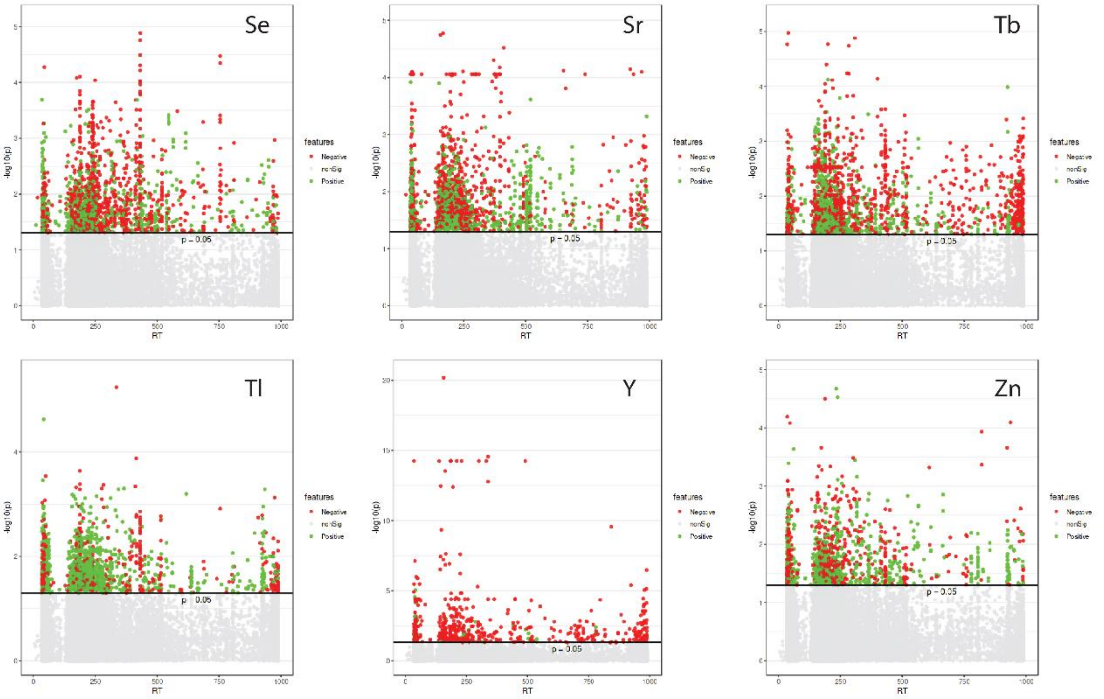

Figure S16. Manhattan plots of metals, Se, Sr, Tb, Tl, Y and Zn in C18 ESI^{-} mode.

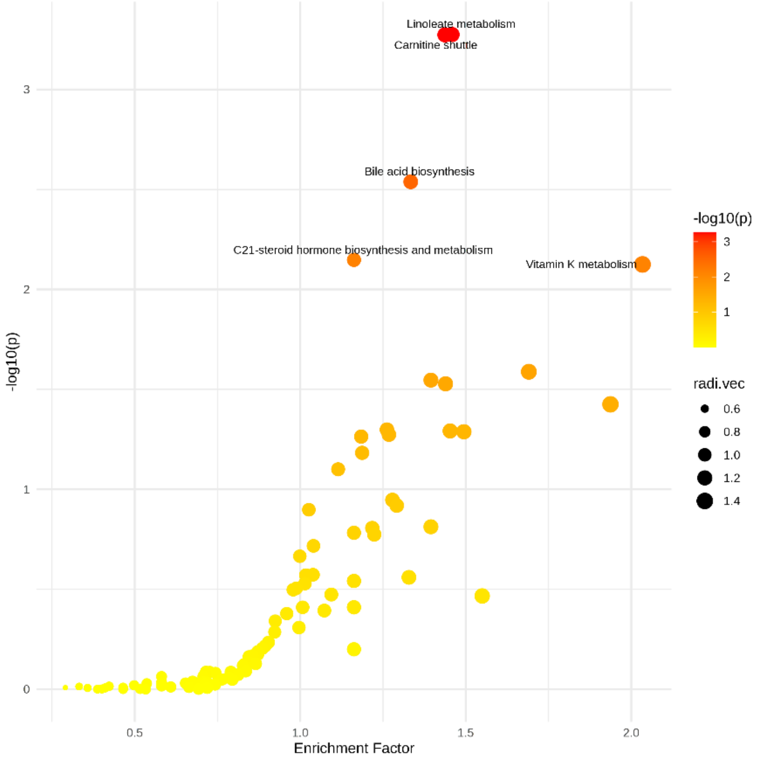

Figure S17. Scatter plot of functional analysis based on HILIC dataset (e-waste workers vs. Control).

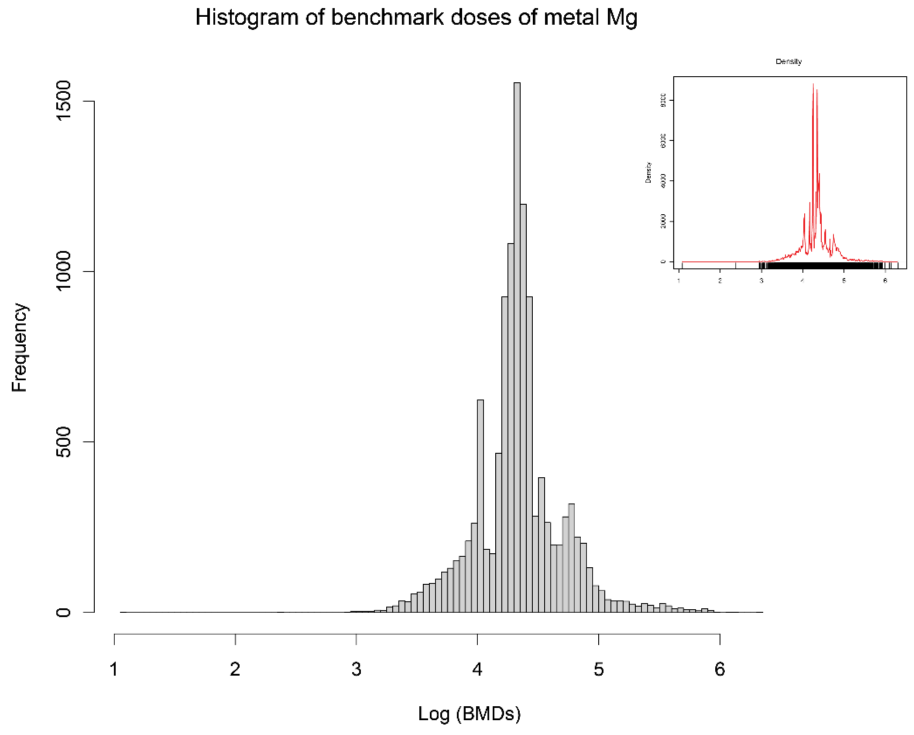

Figure S18. Histogram and density plot of all benchmark doses of metal, Mg.

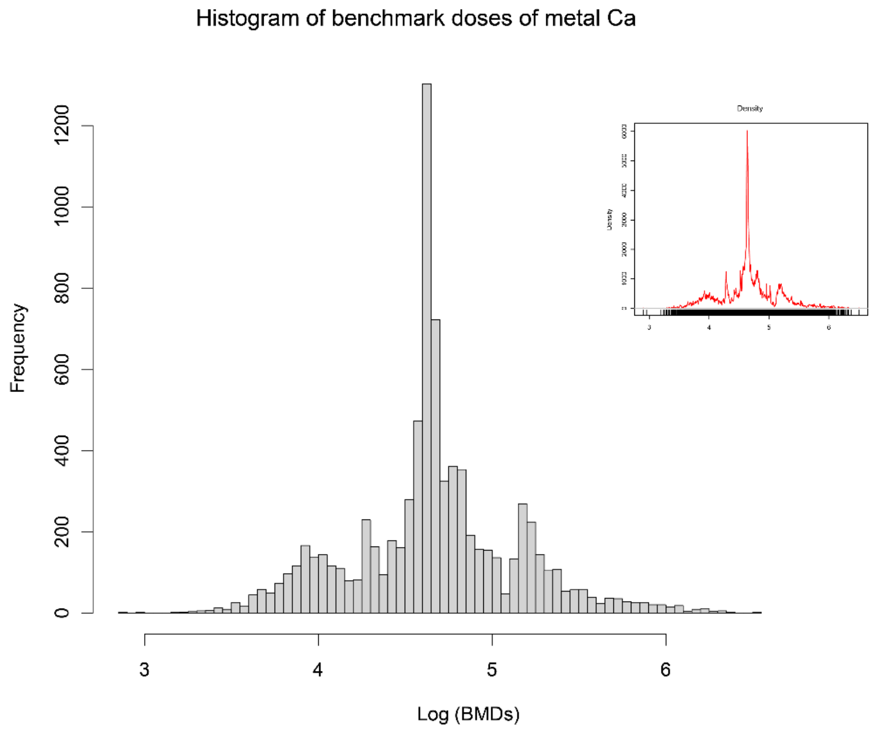

Figure S19. Histogram and density plot of all benchmark doses of metal, Ca.

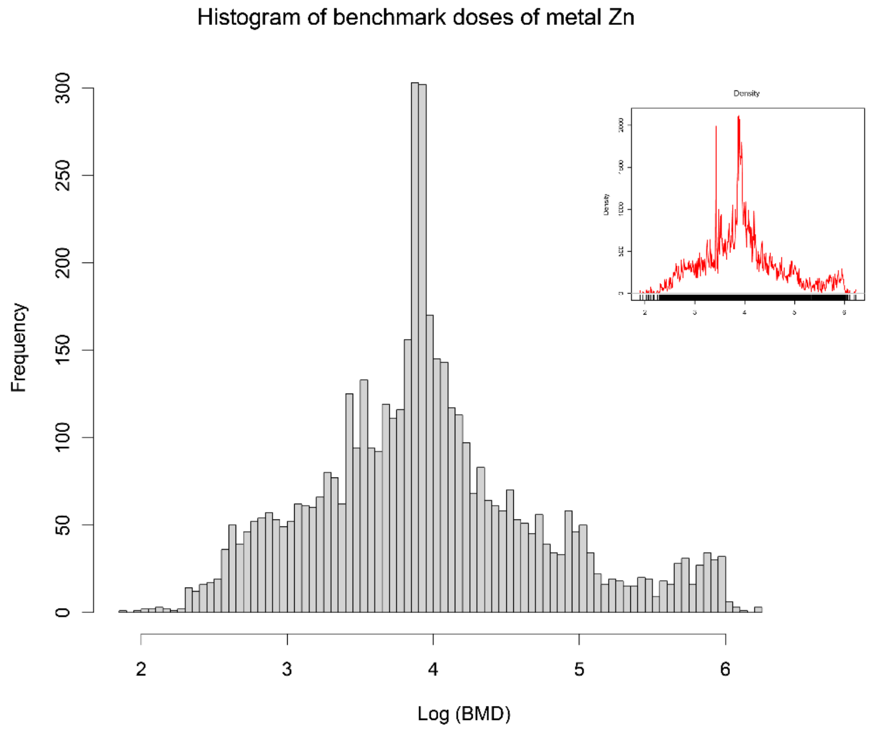

Figure S20. Histogram and density plot of all benchmark doses of metal, Zn.

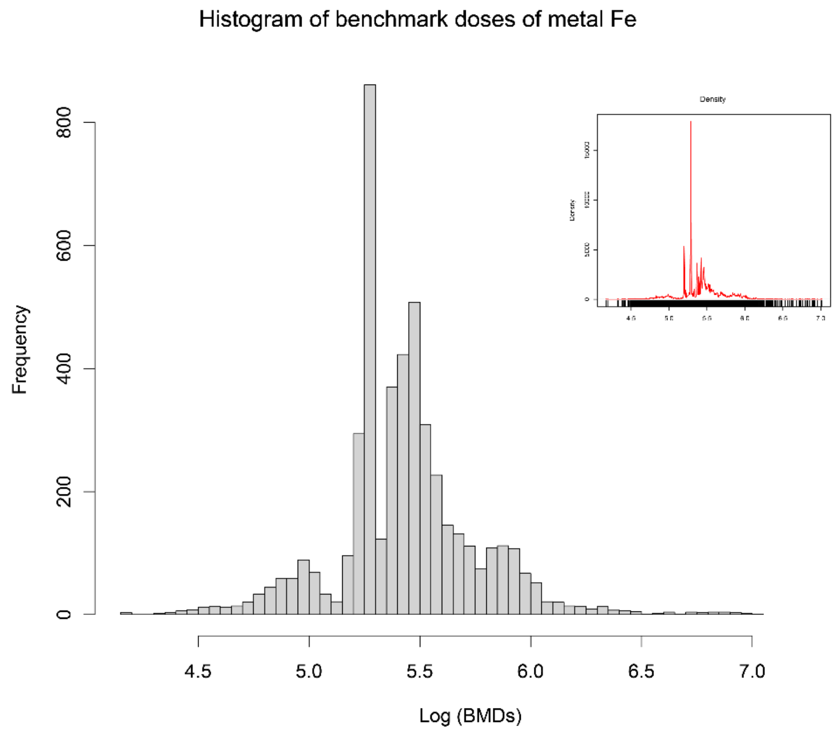

Figure S21. Histogram and density plot of all benchmark doses of metal, Fe.

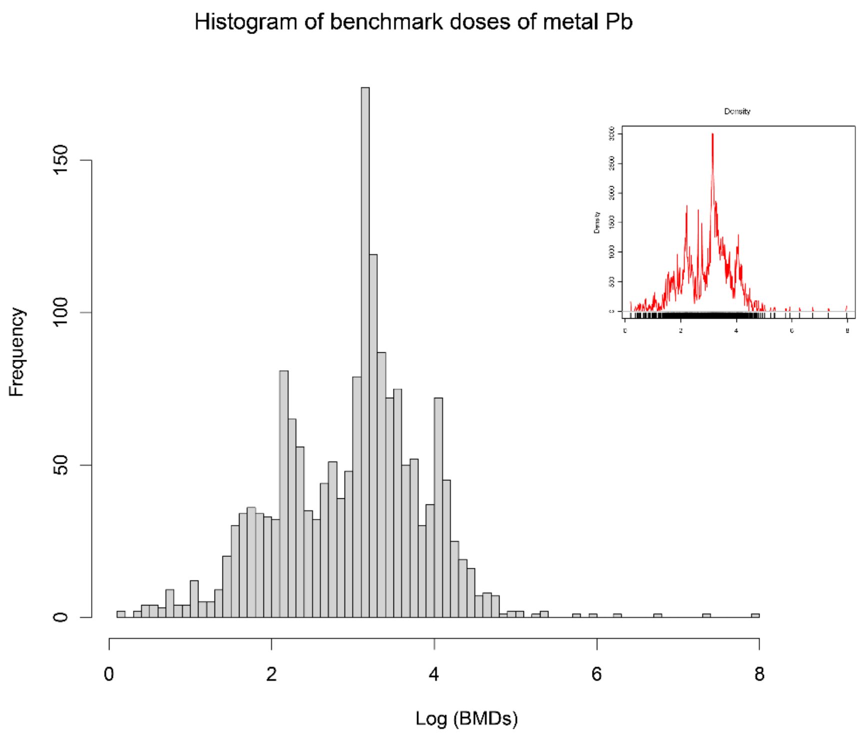

Figure S22. Histogram and density plot of all benchmark doses of metal, Pb.

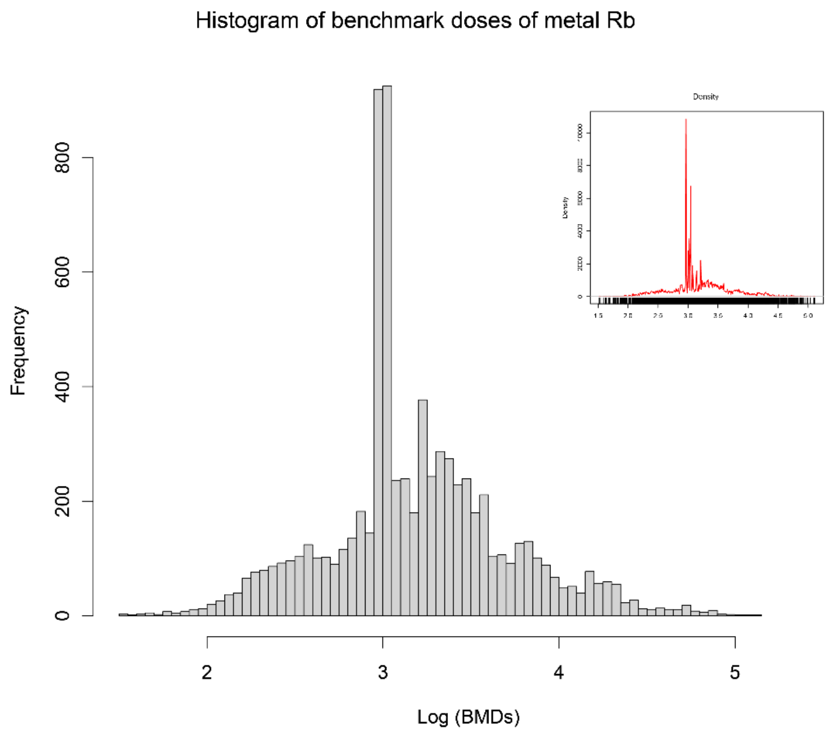

Figure S23. Histogram and density plot of all benchmark doses of metal, Rb.

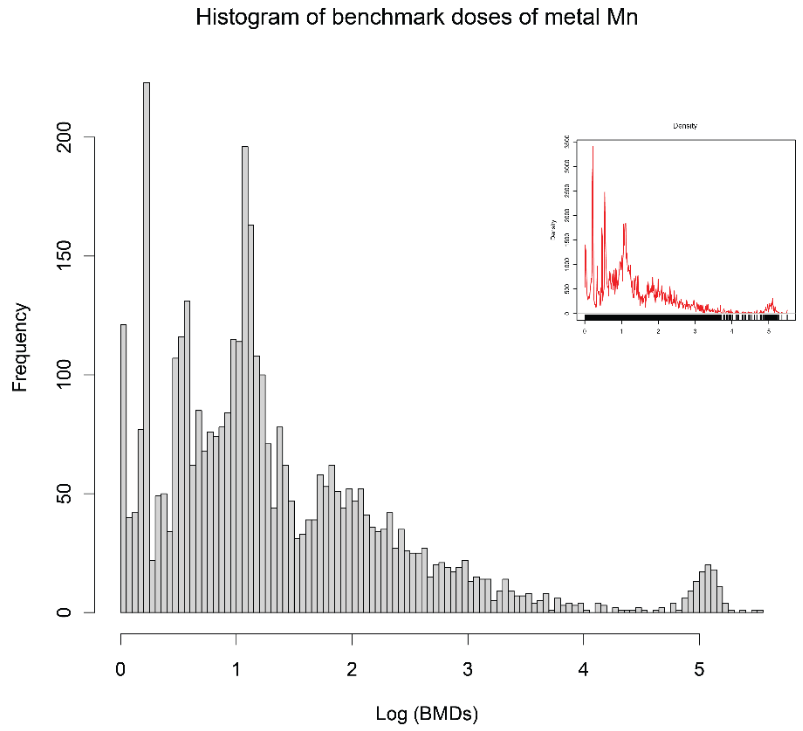

Figure S24. Histogram and density plot of all benchmark doses of metal, Mn.

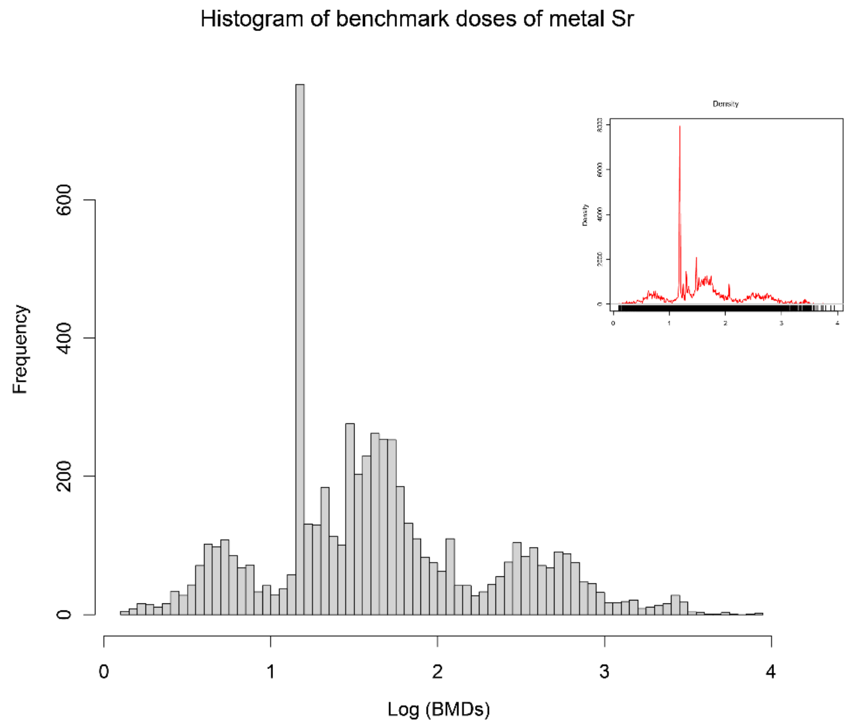

Figure S25. Histogram and density plot of all benchmark doses of metal, Sr.

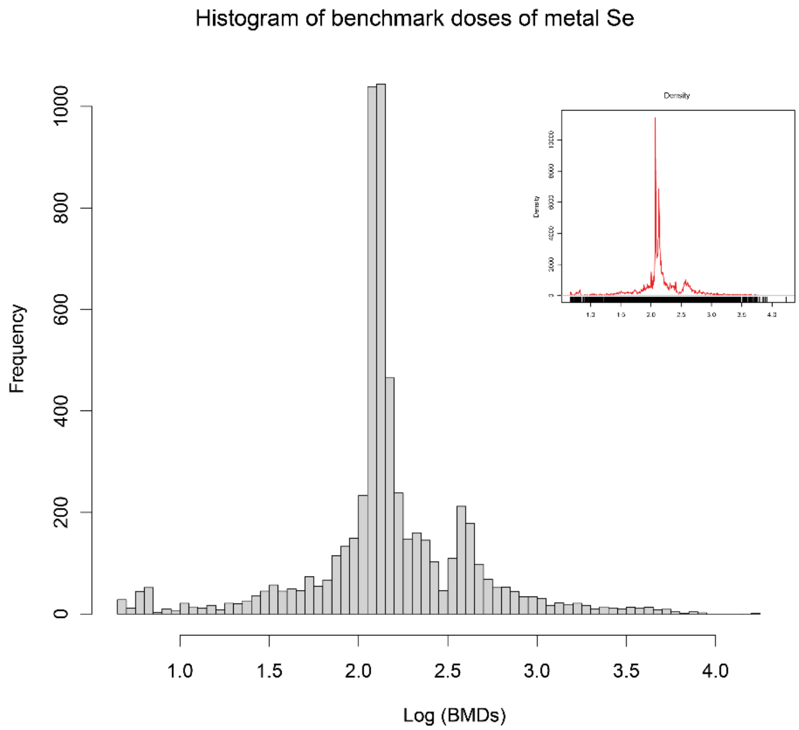

Figure S26. Histogram and density plot of all benchmark doses of metal, Se.

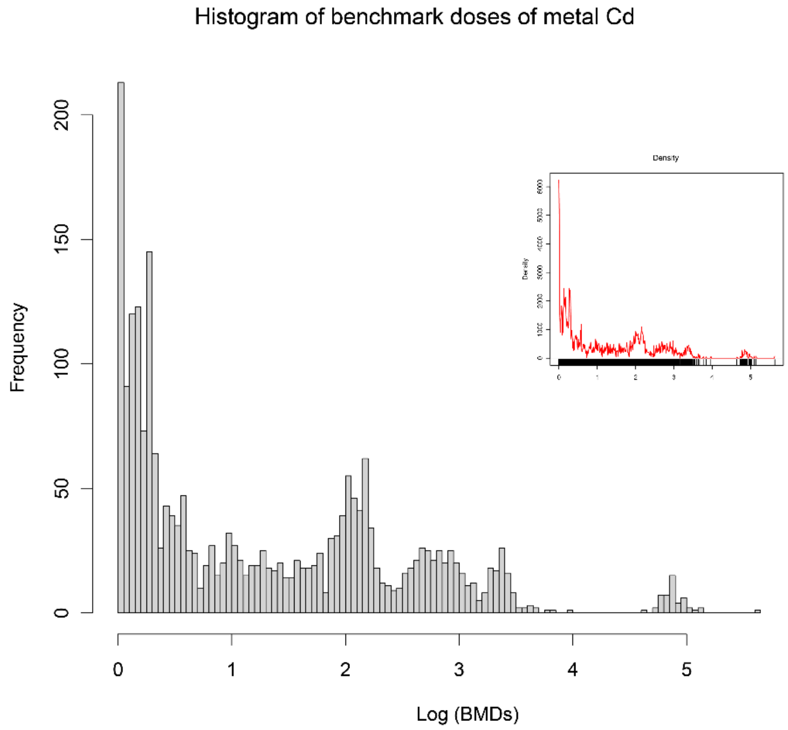

Figure S27. Histogram and density plot of all benchmark doses of metal, Cd.

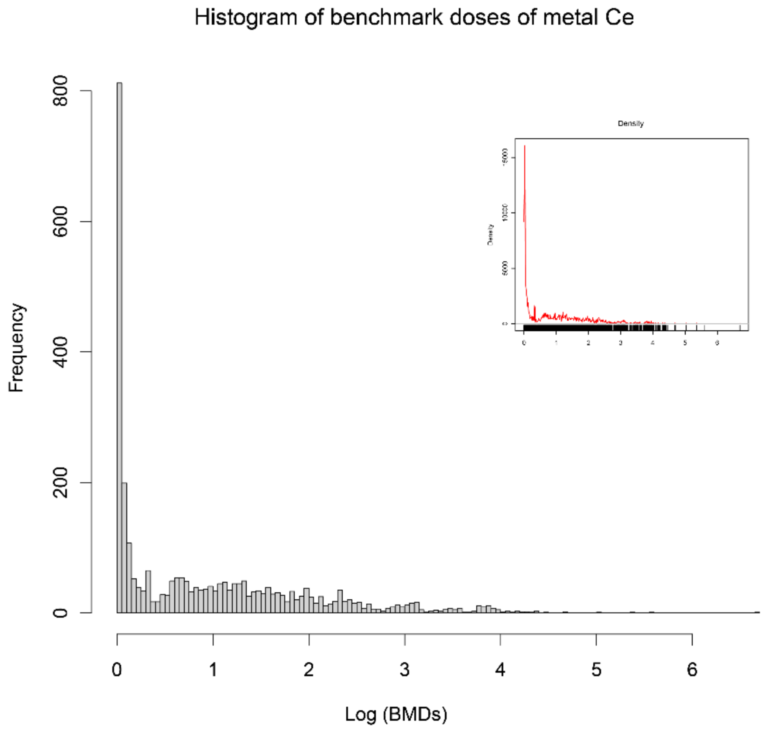

Figure S28. Histogram and density plot of all benchmark doses of metal, Ce.

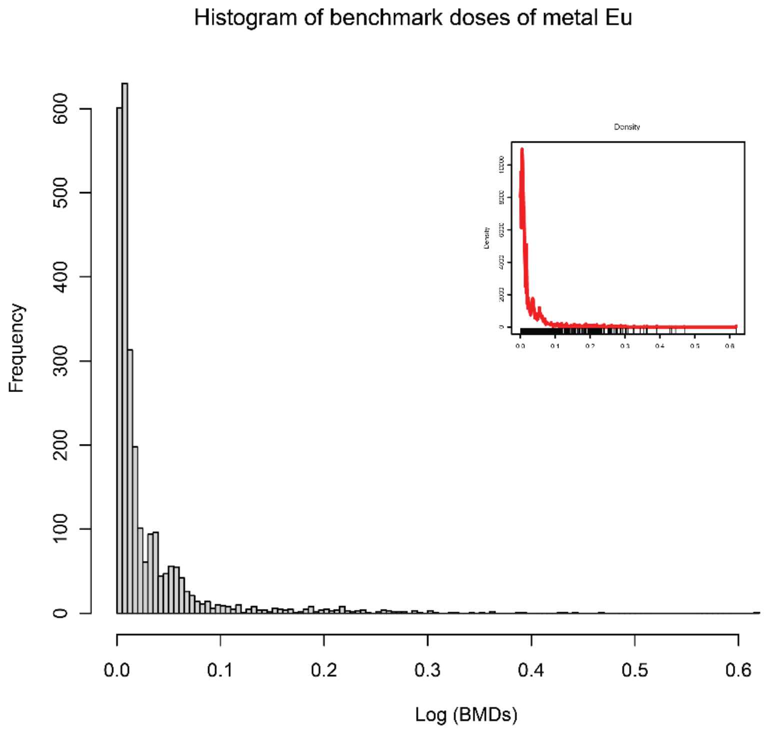

Figure S29. Histogram and density plot of all benchmark doses of metal, Eu.

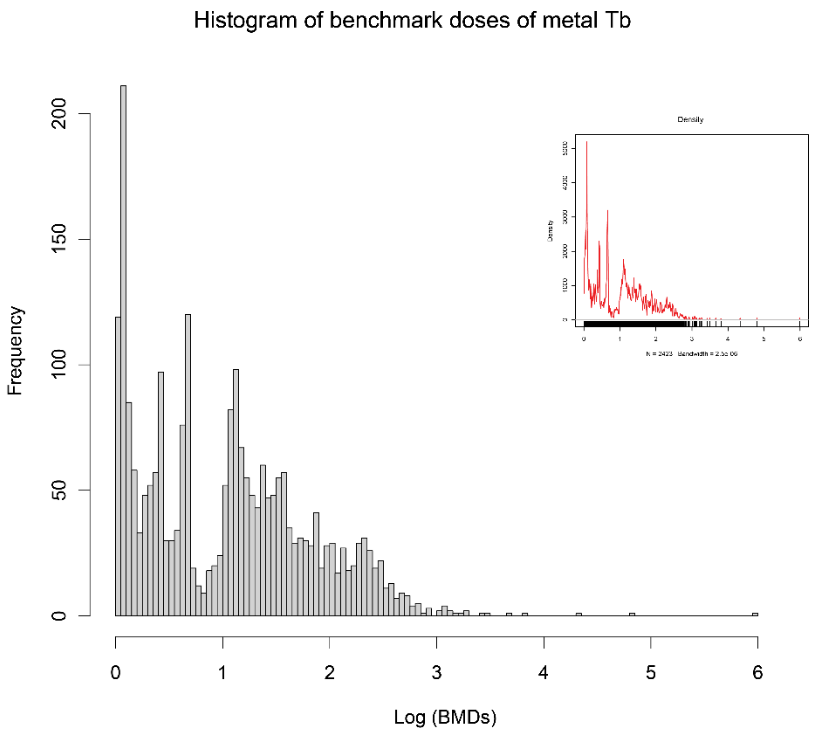

Figure S30. Histogram and density plot of all benchmark doses of metal, Tb.

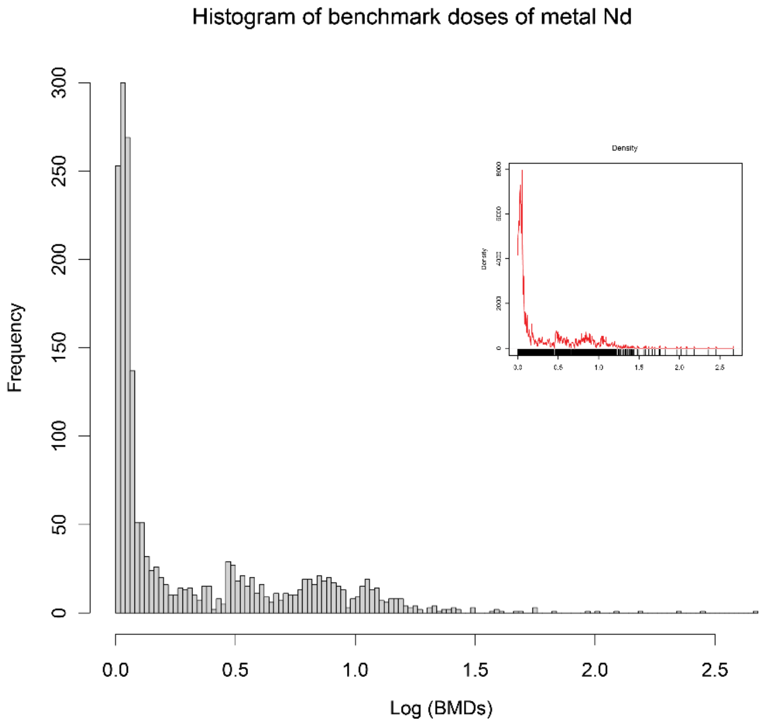

Figure S31. Histogram and density plot of all benchmark doses of metal, Nd.

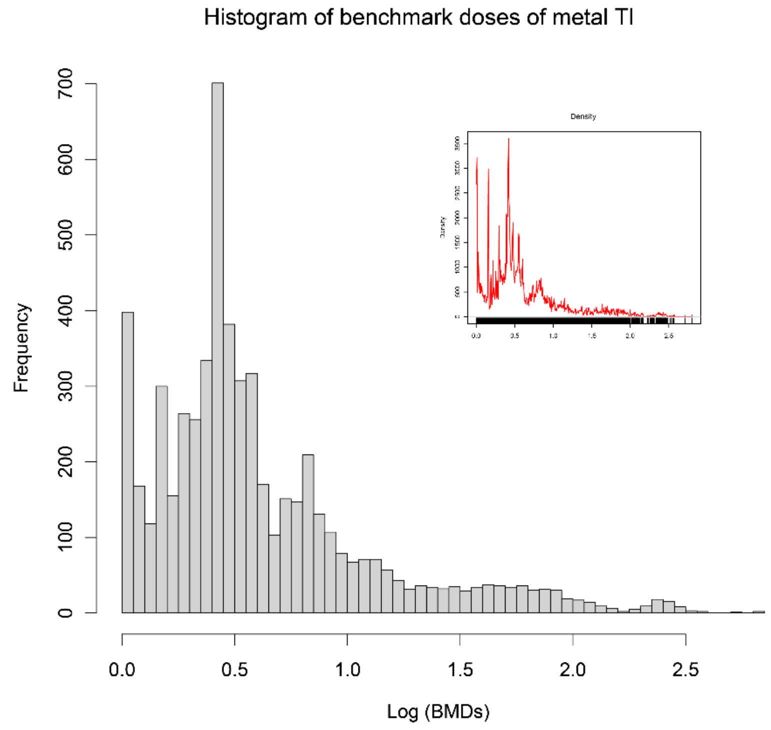

Figure S32. Histogram and density plot of all benchmark doses of metal, Tl.

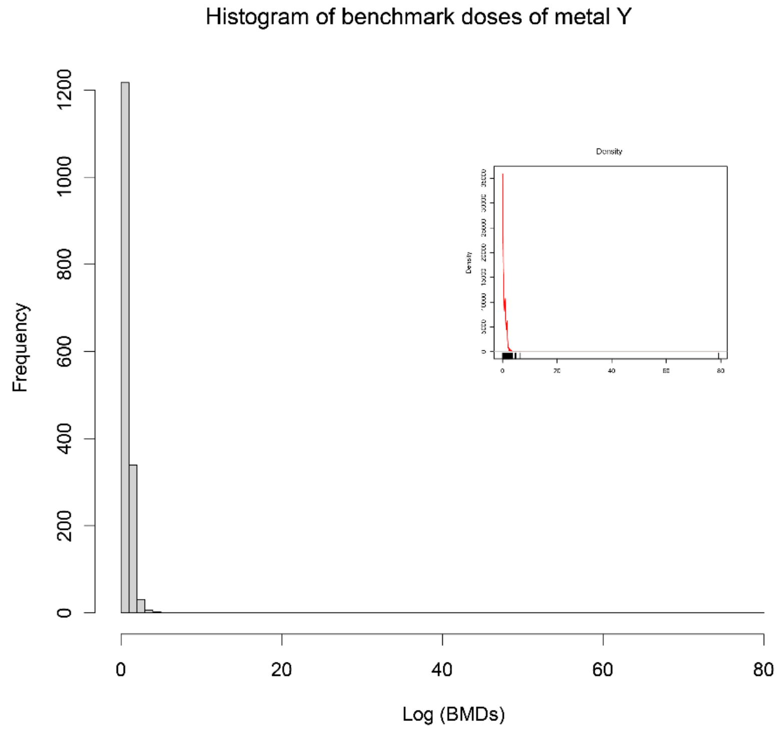

Figure S33. Histogram and density plot of all benchmark doses of metal, Y.

Table S1. Statistics and summary of all detected MS features across different modes and methods

Methods	Modes	Significance	Number
Asari	C18 ESI-	Significant	5371
	C18 ESI+		9203
	HILIC ESI-		7062
	HILIC ESI+		7738
centWave	C18 ESI-		2705
	C18 ESI+		3780
	HILIC ESI-		4229
	HILIC ESI+		5035
Asari	C18 ESI-	Non-Significant	25024
	C18 ESI+		32581
	HILIC ESI-		19425
	HILIC ESI+		16234
centWave	C18 ESI-		11168
	C18 ESI+		10417
	HILIC ESI-		11929
	HILIC ESI+		9810

Table S2. All parameters used for *centWave*

Parameters	C18 modes	HILIC modes
ppm	5	5
Min peakwidth	5	5
Max peakwidth	15	25
mzdiff	0.0015	0.0013
bandwidth	3	2
noise	500	800
prefilter	3, 100	3, 120
minFraction	0.5	0.5

Table S5. Integrated functional analysis results across all metals

Pathways	p_values	FDR	Ratio
Bile acid biosynthesis	1.85E-37	2.00E-35	0.59
C21-steroid hormone biosynthesis and metabolism	5.12E-32	2.76E-30	0.724
Carnitine shuttle	2.28E-23	8.19E-22	0.279
Vitamin E metabolism	2.54E-18	6.86E-17	0.591
Linoleate metabolism	2.43E-16	5.25E-15	0.668
Vitamin K metabolism	1.61E-12	2.91E-11	0.824
Prostaglandin formation from arachidonate	6.65E-11	1.03E-09	0.698
Dynorphin metabolism	7.84E-10	1.06E-08	0.562
Prostaglandin formation from dihomo gama-linoleic acid	4.60E-09	5.52E-08	0.652
Vitamin B6 (pyridoxine) metabolism	6.16E-09	6.65E-08	0.525
Vitamin D3 (cholecalciferol) metabolism	1.16E-07	1.14E-06	0.738
Fatty acid activation	6.32E-07	5.69E-06	0.328
Tryptophan metabolism	8.30E-07	6.90E-06	0.551
Tyrosine metabolism	8.67E-06	6.69E-05	0.512
Drug metabolism - cytochrome P450	0.000231	0.00166	0.661
Ascorbate (Vitamin C) and Aldarate Metabolism	0.000841	0.00568	0.589
Chondroitin sulfate degradation	0.00303	0.0192	0.14
De novo fatty acid biosynthesis	0.00756	0.0454	0.138
Urea cycle/amino group metabolism	0.0321	0.183	0.382
D4&E4-neuroprostanes formation	0.0377	0.204	0.857
Sphingolipid metabolism	0.0478	0.246	0.507
Heparan sulfate degradation	0.054	0.265	0.158
Caffeine metabolism	0.0802	0.377	0.639
Lipoate metabolism	0.15	0.675	0.392
Androgen and estrogen biosynthesis and metabolism	0.162	0.701	0.661
Electron transport chain	0.215	0.894	0.273
Hyaluronan Metabolism	0.42	1	0.503
Leukotriene metabolism	0.443	1	0.481
Putative anti-Inflammatory metabolites formation from EPA	0.486	1	0.761
3-oxo-10R-octadecatrienoate beta-oxidation	0.502	1	0.642
Arginine and Proline Metabolism	0.541	1	0.499
Arachidonic acid metabolism	0.627	1	0.709
Ubiquinone Biosynthesis	0.634	1	0.656
Alkaloid biosynthesis II	0.676	1	0.403
Fatty Acid Metabolism	0.822	1	0.169
TCA cycle	0.835	1	0.387
Vitamin B1 (thiamin) metabolism	0.891	1	0.357
Limonene and pinene degradation	0.913	1	0.592
1- and 2-Methylnaphthalene degradation	0.935	1	0.758
Glycerophospholipid metabolism	0.952	1	0.232
Alanine and Aspartate Metabolism	0.962	1	0.371
Pentose and Glucuronate Interconversions	0.964	1	0.794

Glycosphingolipid metabolism	0.969	1	0.33
Aspartate and asparagine metabolism	0.974	1	0.373
Histidine metabolism	0.98	1	0.453
Butanoate metabolism	0.985	1	0.408
Lysine metabolism	0.985	1	0.327
Nitrogen metabolism	0.987	1	0.422
C5-Branched dibasic acid metabolism	0.993	1	0.268
Biopterin metabolism	0.993	1	0.544
Propanoate metabolism	0.994	1	0.232
Porphyrin metabolism	0.995	1	0.318
Vitamin A (retinol) metabolism	0.995	1	0.497
Beta-Alanine metabolism	0.997	1	0.328
Sialic acid metabolism	0.997	1	0.227
Squalene and cholesterol biosynthesis	0.997	1	0.403
Glutamate metabolism	0.998	1	0.515
Parathio degradation	0.998	1	0.557
Benzoate degradation via CoA ligation	0.999	1	0.375
Mono-unsaturated fatty acid beta-oxidation	0.999	1	0.0727
Glycine, serine, alanine and threonine metabolism	1	1	0.366
Glycosphingolipid biosynthesis - ganglioseries	1	1	0.149
Polyunsaturated fatty acid biosynthesis	1	1	0.0629
Omega-3 fatty acid metabolism	1	1	0.15
Omega-6 fatty acid metabolism	1	1	0.0525
Pyruvate Metabolism	1	1	0.3
Methionine and cysteine metabolism	1	1	0.281
CoA Catabolism	1	1	0.401
Drug metabolism - other enzymes	1	1	0.441
Carbon fixation	1	1	0.594
Hexose phosphorylation	1	1	0.668
Vitamin B2 (riboflavin) metabolism	1	1	0.316
Glutathione Metabolism	1	1	0.336
Glycolysis and Gluconeogenesis	1	1	0.435
N-Glycan Degradation	1	1	0.316
Pentose phosphate pathway	1	1	0.696
Phosphatidylinositol phosphate metabolism	1	1	0.358
Fructose and mannose metabolism	1	1	0.601
Vitamin B5 - CoA biosynthesis from pantothenate	1	1	0.435
Glycosylphosphatidylinositol (GPI)-anchor biosynthesis	1	1	0.282
Proteoglycan biosynthesis	1	1	0.143
Vitamin H (biotin) metabolism	1	1	0.624
Galactose metabolism	1	1	0.625
Selenoamino acid metabolism	1	1	0.267
Keratan sulfate biosynthesis	1	1	0.0593
Glyoxylate and Dicarboxylate Metabolism	1	1	0.301

Di-unsaturated fatty acid beta-oxidation	1	1	0.0781
Phytanic acid peroxisomal oxidation	1	1	0.0907
Keratan sulfate degradation	1	1	0.0715
Valine, leucine and isoleucine degradation	1	1	0.248
Glycosphingolipid biosynthesis - globoseries	1	1	0.328
Starch and Sucrose Metabolism	1	1	0.342
Fatty acid oxidation	1	1	0.0597
Fatty acid oxidation, peroxisome	1	1	0.0756
Aminosugars metabolism	1	1	0.303
Xenobiotics metabolism	1	1	0.374
Purine metabolism	1	1	0.462
O-Glycan biosynthesis	1	1	0.198
Vitamin B9 (folate) metabolism	1	1	0.328
Nucleotide Sugar Metabolism	1	1	0.484
Vitamin B3 (nicotinate and nicotinamide) metabolism	1	1	0.506
Vitamin B12 (cyanocobalamin) metabolism	1	1	0.258
Pyrimidine metabolism	1	1	0.463
Glycosphingolipid biosynthesis - lactoseries	1	1	0.226
Blood Group Biosynthesis	1	1	0.0777
Glycosphingolipid biosynthesis - neolactoseries	1	1	0.214
N-Glycan biosynthesis	1	1	0.224
Saturated fatty acids beta-oxidation	1	1	0.0824

Table S6. Summary of all MS features fitting dose-response curves.

**Metal**	**Number of MS features fitting the dose-response curve**	**Number of all Linear regressed MS features**	**Ratio (%)**
Mg	11679	16982	68.77282
Ca	8146	11448	71.15653
Cu	10749	15302	70.24572
Zn	4779	8083	59.12409
Fe	4755	7525	63.18937
Se	5903	11303	52.22507
Mn	3963	7123	55.63667
Cd	2320	6069	38.22706
Pb	1721	3185	54.03454
Rb	7664	11135	68.82802
Sr	5948	9555	62.25013
Y	1597	2742	58.24216
La	2423	4669	51.89548
Ce	2907	6134	47.39159
Nd	1862	4229	44.02932
Eu	2607	4532	57.52427
Tb	6305	10852	58.09989
BTl	5701	9473	60.18157
